# Supplementary material for: Detection and evolutionary characterization of arboviruses in mosquitoes and biting midges on Hainan Island, China, 2019–2023
Source: PLoS Negl Trop Dis. 2024 Oct 31;18(10):e0012642. doi: 10.1371/journal.pntd.0012642 (PMC11556698; doi:10.1371/journal.pntd.0012642)
Supplement: S1 Table — (DOCX) [file pntd.0012642.s001.docx]

S1 Table. Primers used in the heminested RT-PCR or RT-PCR assay for detecting arboviruses

|  | Primer | Sequence (5′−3′) | Length (bp) | Annealing  temperature (℃) | Ref |
| --- | --- | --- | --- | --- | --- |
| Flavivirus | XF-F1 | AACATGATGGGVAARMGWGARAA | 263 | 52 | [15] |
|  | XF-R | GTRTCCCANCCDGCDGTRTCATCNGC |  |  |  |
|  | XF-F2 | AARGGMAGYMGNGCHATHTGGT | 215 | 54 |  |
|  | XF-R | GTRTCCCANCCDGCDGTRTCATCNGC |  |  |  |
| Alphaviruses | XA-F1 | AGAGCRTTYTCGCATCTRGCYAK | 433 | 54 | [16] |
|  | XA-R | ACATGAACKGRGTKGTGTCRAASCCWAYCC |  |  |  |
|  | XA-F2 | TGCCCBRTGCGBAGYSCVGAAGAYCC | 310 | 60 |  |
|  | XA-R | ACATGAACKGRGTKGTGTCRAASCCWAYCC |  |  |  |
| Orthobunyavirus | BUP | ATGACTGAGTTGGAGTTTGATGTCGC | 251 | 55 | [13] |
|  | BDW | TGTTCCTGTTGCCAGGAAAAT |  |  |  |
| Bluetongue virus  (BTV) | BTV-S1-F | GGCAACACTCCCTTTGGGAT | 426 | 55 |  |
|  | BTV-S1-R | TTCACGGGTTGGCGGAATTA |  |  |  |

*N=A+G+C+T, V=G+A+C, R=A+G, M=A+C, W=A+T, Y=C+T, H=A+T+C, D=G+A+T,K=G+T，B=G+C+T，S=G+C，V=G+A+C.
