# Supplementary material for: Detection and evolutionary characterization of arboviruses in mosquitoes and biting midges on Hainan Island, China, 2019–2023
Source: PLoS Negl Trop Dis. 2024 Oct 31;18(10):e0012642. doi: 10.1371/journal.pntd.0012642 (PMC11556698; doi:10.1371/journal.pntd.0012642)
Supplement: S2 Table — (DOCX) [file pntd.0012642.s002.docx]

S2 Table. Geographic and Species information on Sample Collection in Hainan Island 2019-2023

| NO. | Sample ID | Collection date | Location* | East longitude | Northern  latitude | Habitat types | Genus | Species | Pools | Number of mosquitoes or midges |
| --- | --- | --- | --- | --- | --- | --- | --- | --- | --- | --- |
| 1 | 2019-HNLS-An | 2019.08.08-09 | LS | E110°03′56″ | N18°36′59″ | Cattle farm | Anopheles | *Anopheles.sp* | 1 | 20 |
| 2 | 2019-HNLS-Mu | 2019.08.08-09 | LS | E110°03′56″ | N18°36′59″ | Cattle farm | Mansonia | *Mansonia uniformi*s | 1 | 25 |
| 3 | 2019-HNLS-Ct | 2019.08.08-09 | LS | E110°03′56″ | N18°36′59″ | Cattle farm | Culex | *Culex tritaeniorhynchus* | 6 | 315 |
| 4 | 2019-HNLD-Cg | 2019.10.9-10 | LD | E109°11′09″ | N18°45′03″ | Peasant household | Culex | *Culex gelidus* | 1 | 28 |
| 5 | 2019-HNLD-Ct | 2019.10.9-10 | LD | E109°11′09″ | N18°45′03″ | Peasant household | Culex | *Culex tritaeniorhynchus* | 4 | 175 |
| 6 | 2019-HNCJ-Ae | 2019.08.16-17 | CJ | E109°09′12″ | N19°00′46″ | Vegetable field | Aedes | *Aedes albopictus* | 1 | 15 |
| 7 | 2019-HNCJ-Ct | 2019.08.16-17 | CJ | E109°09′12″ | N19°00′46″ | Vegetable field | Culex | *Culex tritaeniorhynchus* | 1 | 30 |
| 8 | 2019-HNCJ-An | 2019.08.16-17 | CJ | E109°09′12″ | N19°00′46″ | Vegetable field | Anopheles | *Anopheles.sp* | 1 | 20 |
| 9 | 2021-HNQZ-Ae | 2021.06.29-07.01 | QZ | E109°49′54″ | N19°01′45″ | Peasant household | Aedes | *Aedes albopictus* | 25 | 725 |
| 10 | 2021-HNQZ-As | 2021.06.29-07.01 | QZ | E109°43′49″ | N19°02′10″ | Cattle farm | Armigeres | *Armigeres subalbatus* | 11 | 260 |
| 11 | 2021-HNQZ-Ct | 2021.06.29-07.01 | QZ | E109°43′49″ | N19°02′10″ | Cattle farm | Culex | *Culex tritaeniorhynchus* | 25 | 750 |
| 12 | 2021-HNQZ-Cg | 2021.06.29-07.01 | QZ | E109°43′49″ | N19°02′10″ | Cattle farm | Culex | *Culex gelidus* | 7 | 184 |
| 13 | 2021-HNSY-Ae | 2021.07.19-21 | SY | E109°30′43″ | N18°17′50″ | School campus | Aedes | *Aedes albopictus* | 20 | 589 |
| 14 | 2021-HNSY-Ct | 2021.07.19-21 | SY | E109°27′30″ | N18°19′28″ | Cattle farm | Culex | *Culex tritaeniorhynchus* | 29 | 870 |
| 15 | 2021-HNHK-Cg | 2021.07.06 | HK | E110°21′44″ | N19°54′44″ | Cattle farm | Culex | *Culex gelidus* | 11 | 539 |
| 16 | 2021-HNHK-Ct | 2021.07.06 | HK | E110°21′44″ | N19°54′44″ | Cattle farm | Culex | *Culex tritaeniorhynchus* | 5 | 250 |
| 17 | 2021-HNHK-As | 2021.07.06 | HK | E110°21′44″ | N19°54′44″ | Cattle farm | Armigeres | *Armigeres subalbatus* | 6 | 250 |
| 18 | 2021-HNHK-A-male | 2021.07.06 | HK | E110°21′44″ | N19°54′44″ | Cattle farm | Armigeres | *Armigeres subalbatus* | 1 | 33 |
| 19 | 2022-HNQZ-Ae | 2022.07.20-22 | QZ | E109°51′45″ | N19°01′59″ | Peasant household | Aedes | *Aedes albopictus* | 27 | 1620 |
| 20 | 2022-HNQZ-As | 2022.07.20-22 | QZ | E109°42′50″ | N19°05′07″ | Cattle farm | Armigeres | *Armigeres subalbatus* | 1 | 40 |
| 21 | 2022-HNQZ-Ct | 2022.07.20-22 | QZ | E109°42′50″ | N19°05′07″ | Cattle farm | Culex | *Culex tritaeniorhynchus* | 19 | 1140 |
| 22 | 2022-HNQZ-Cu | 2022.07.20-22 | QZ | E109°42′50″ | N19°05′07″ | Cattle farm | Culex | *Culex.sp* | 9 | 540 |
| 23 | 2022-HNQZ-An | 2022.07.20-22 | QZ | E109°42′50″ | N19°05′07″ | Cattle farm | Anopheles | *Anopheles.sp* | 1 | 30 |
| 24 | 2022-HNSY-Cu | 2022.04.25-26 | SY | E109°30′43″ | N18°17′50″ | Cattle farm | Culex | *Culex.sp* | 33 | 1980 |
| 25 | 2022-HNSY-Ae | 2022.04.25-26 | SY | E109°30′43″ | N18°17′50″ | School campus | Aedes | *Aedes albopictus* | 3 | 180 |
| 26 | 2022-HNSY-As | 2022.04.25-26 | SY | E109°30′43″ | N18°17′50″ | Cattle farm | Armigeres | *Armigeres subalbatus* | 2 | 100 |
| 27 | 2022-HNDZ-As | 2022.03.22-24 | DZ | E109°23'03″ | N19°31'05″ | Cattle farm | Armigeres | *Armigeres subalbatus* | 11 | 387 |
| 28 | 2022-HNDZ-Cu | 2022.03.22-24 | DZ | E109°23'03″ | N19°31'05″ | Cattle farm | Culex | *Culex.sp* | 4 | 247 |
| 29 | 2022-HNDZ-Cq | 2022.03.22-24 | DZ | E109°23'03″ | N19°31'05″ | Cattle farm | Culex | *Culex quinquefasciatus* | 1 | 61 |
| 30 | 2022-HNLS-Cu | 2022.06.16-17 | LS | E110°03′56″ | N18°36′59″ | Cattle farm | Culex | *Culex.sp* | 47 | 2940 |
| 31 | 2022-HNLS-Ae | 2022.06.16-17 | LS | E109°59′04″ | N18°26′23″ | Peasant household | Aedes | *Aedes albopictus* | 3 | 270 |
| 32 | 2022-HNLS-As | 2022.06.16-17 | LS | E109°59′04″ | N18°26′23″ | Cattle farm | Armigeres | *Armigeres subalbatus* | 2 | 90 |
| 33 | 2022-HNLS-An | 2022.06.16-17 | LS | E109°59′04″ | N18°26′23″ | Cattle farm | Anopheles | *Anopheles.sp* | 1 | 50 |
| 34 | 2022-HNTC-As | 2022.08.01-03 | TC | E110°06'18″ | N19°22'17″ | Cattle farm | Armigeres | *Armigeres subalbatus* | 24 | 1048 |
| 35 | 2022-HNTC-Ae | 2022.08.01-03 | TC | E110°05'46″ | N19°20'19″ | Flowerbed | Aedes | *Aedes albopictus* | 14 | 626 |
| 36 | 2022-HNTC-Ct | 2022.08.01-03 | TC | E110°06'18″ | N19°22'17″ | Cattle farm | Culex | *Culex tritaeniorhynchus* | 10 | 457 |
| 37 | 2022-HNTC-Cg | 2022.08.01-03 | TC | E110°06'18″ | N19°22'17″ | Cattle farm | Culex | *Culex gelidus* | 4 | 190 |
| 38 | 2022-HNTC-An | 2022.08.01-03 | TC | E110°06'18″ | N19°22'17″ | Cattle farm | Anopheles | *Anopheles.sp* | 1 | 24 |
| 39 | 2022-HNWN-Cu | 2022.04.25-27 | WN | E110°11′37″ | N18°41′32″ | Cattle farm | Culex | *Culex.sp* | 24 | 2242 |
| 40 | 2022-HNWN-An | 2022.04.25-27 | WN | E110°11′37″ | N18°41′32″ | Cattle farm | Anopheles | *Anopheles.sp* | 2 | 120 |
| 41 | 2022-HNWN-As | 2022.04.25-27 | WN | E110°11′37″ | N18°41′32″ | Cattle farm | Armigeres | *Armigeres subalbatus* | 3 | 72 |
| 42 | 2022-HNWZS-Ae | 2022.09.21-23 | WZS | E109°30′04″ | N18°47′53″ | Betel palm tree | Aedes | *Aedes albopictus* | 7 | 161 |
| 43 | 2022-HNWZS-Cu | 2022.09.21-23 | WZS | E109°30′04″ | N18°47′53″ | Pigsty | Culex | *Culex.sp* | 1 | 20 |
| 44 | 2022-HNWZS-Ct | 2022.09.21-23 | WZS | E109°30′04″ | N18°47′53″ | Pigsty | Culex | *Culex tritaeniorhynchus* | 1 | 41 |
| 45 | 2022-HNWZS-As | 2022.09.21-23 | WZS | E109°30′04″ | N18°47′53″ | Pigsty | Armigeres | *Armigeres subalbatus* | 2 | 30 |
| 46 | 2022-HNWZS-An | 2022.09.21-23 | WZS | E109°30′04″ | N18°47′53″ | Pigsty | Anopheles | *Anopheles.sp* | 1 | 15 |
| 47 | 2023-HNQZ-As | 2023.05.18 | QZ | E109°42′37″ | N19°05′31″ | Cattle farm | Armigeres | *Armigeres subalbatus* | 2 | 81 |
| 48 | 2023-HNQZ-Ae | 2023.07.20 | QZ | E109°39′40″ | N19°01′49″ | Coconut grove | Aedes | *Aedes albopictus* | 4 | 138 |
| 49 | 2023-HNQZ-Cg | 2023.07.20 | QZ | E109°42′37″ | N19°05′31″ | Cattle farm | Culex | *Culex gelidus* | 2 | 14 |
| 50 | 2023-HNQZ-Ct | 2023.07.20 | QZ | E109°42′37″ | N19°05′31″ | Cattle farm | Culex | *Culex tritaeniorhynchus* | 4 | 220 |
| 51 | 2023-HNQZ-Cq | 2023.05.18 | QZ | E109°42′37″ | N19°05′31″ | Cattle farm | Culex | *Culex quinquefasciatus* | 1 | 7 |
| 52 | 2023-HNQZ-An | 2023.05.18 | QZ | E109°42′37″ | N19°05′31″ | Cattle farm | Anopheles | *Anopheles.sp* | 5 | 184 |
| 53 | 2023-HNSY-An | 2023.07.13-14 | SY | E109°27′27″ | N18°19′30″ | Cattle farm | Anopheles | *Anopheles.sp* | 1 | 22 |
| 54 | 2023-HNSY-As | 2023.07.13-14 | SY | E109°27′27″ | N18°19′30″ | Cattle farm | Armigeres | *Armigeres subalbatus* | 3 | 34 |
| 55 | 2023-HNSY-Cg | 2023.07.13-14 | SY | E109°27′27″ | N18°19′30″ | Cattle farm | Culex | *Culex gelidus* | 1 | 17 |
| 56 | 2023-HNSY-Ct | 2023.07.13-14 | SY | E109°27′27″ | N18°19′30″ | Cattle farm | Culex | *Culex tritaeniorhynchus* | 22 | 1448 |
| 57 | 2023-HNSY-Ae | 2023.07.13-14 | SY | E109°30′41″ | N18°17′52″ | School campus | Aedes | *Aedes albopictus* | 6 | 341 |
| 58 | 2023-HNSY-Ae-male | 2023.07.13-14 | SY | E109°30′41″ | N18°17′52″ | School campus | Aedes | *Aedes albopictus* | 1 | 35 |
| 59 | 2023-HNDZ-As | 2023.04.13-14 | DZ | E109°44′46″ | N19°25′52″ | Pigsty,Cattle farm | Armigeres | *Armigeres subalbatus* | 2 | 55 |
| 60 | 2023-HNDZ-An | 2023.04.13-14 | DZ | E109°19′46″ | N19°19′46″ | Pigsty,Cattle farm | Anopheles | *Anopheles.sp* | 2 | 15 |
| 61 | 2023-HNDZ-Ct | 2023.04.13-14 | DZ | E109°44′46″ | N19°25′52″ | Pigsty,Cattle farm | Culex | *Culex tritaeniorhynchus* | 2 | 36 |
| 62 | 2023-HNDZ-Mu | 2023.04.13-14 | DZ | E109°44′46″ | N19°25′52″ | Pigsty,Cattle farm | Mansonia | *Mansonia uniformi*s | 1 | 1 |
| 63 | 2023-HNLS-Cg | 2023.07.24-25 | LS | E110°03′13″ | N18°37′03″ | Cattle farm | Culex | *Culex gelidus* | 1 | 17 |
| 64 | 2023-HNLS-An | 2023.07.24-25 | LS | E110°03′13″ | N18°37′03″ | Cattle farm | Anopheles | *Anopheles.sp* | 1 | 55 |
| 65 | 2023-HNLS-Ae | 2023.07.24-25 | LS | E109°59′04″ | N18°26′22″ | Coconut grove | Aedes | *Aedes albopictus* | 7 | 272 |
| 66 | 2023-HNLS-As | 2023.07.24-25 | LS | E110°03′13″ | N18°37′03″ | Cattle farm | Armigeres | *Armigeres subalbatus* | 4 | 95 |
| 67 | 2023-HNLS-Cq | 2023.07.24-25 | LS | E110°03′13″ | N18°37′03″ | Cattle farm | Culex | *Culex quinquefasciatus* | 3 | 15 |
| 68 | 2023-HNLS-Ct | 2023.07.24-25 | LS | E110°03′13″ | N18°37′03″ | Cattle farm | Culex | *Culex tritaeniorhynchus* | 16 | 897 |
| 69 | 2023-HNLS-Mu | 2023.07.24-25 | LS | E110°03′13″ | N18°37′03″ | Cattle farm | Mansonia | *Mansonia uniformi*s | 2 | 92 |
| 70 | 2023-HNTC-Ae | 2023.06.14 | TC | E110°05′23″ | N19°12′04″ | Rubber Grove,Cattle | Aedes | *Aedes albopictus* | 1 | 32 |
| 71 | 2023-HNTC-An | 2023.06.14 | TC | E110°05′23″ | N19°12′04″ | Rubber Grove,Cattle | Anopheles | *Anopheles.sp* | 1 | 11 |
| 72 | 2023-HNTC-Cu | 2023.06.14 | TC | E110°05′23″ | N19°12′04″ | Rubber Grove,Cattle | Culex | *Culex.sp* | 8 | 742 |
| 73 | 2023-HNTC-As | 2023.06.14 | TC | E110°05′23″ | N19°12′04″ | Rubber Grove,Cattle | Armigeres | *Armigeres subalbatus* | 2 | 94 |
| 74 | 2023-HNWN-Cg | 2023.08.02-04 | WN | E110°16′15″ | N18°48′08″ | Pigsty,Cattle farm | Culex | *Culex gelidus* | 12 | 714 |
| 75 | 2023-HNWN-Cb | 2023.08.02-04 | WN | E110°16′15″ | N18°48′08″ | Pigsty,Cattle farm | Culex | *Culex bitaeniorhynchus* | 1 | 20 |
| 76 | 2023-HNWN-Al | 2023.08.02-04 | WN | E110°16′15″ | N18°48′08″ | Pigsty,Cattle farm | Aedes | *Aedes lineatopennis* | 1 | 2 |
| 77 | 2023-HNWN-As | 2023.08.02-04 | WN | E110°16′15″ | N18°48′08″ | Pigsty,Cattle farm | Armigeres | *Armigeres subalbatus* | 2 | 81 |
| 78 | 2023-HNWN-Ct | 2023.08.02-04 | WN | E110°16′15″ | N18°48′08″ | Pigsty,Cattle farm | Culex | *Culex tritaeniorhynchus* | 22 | 1269 |
| 79 | 2023-HNWN-Ae | 2023.08.02-04 | WN | E110°21′39″ | N18°47′00″ | Peasant household | Aedes | *Aedes albopictus* | 5 | 232 |
| 80 | 2023-HNWC-As | 2023.04.18-19 | WC | E110°35′06″ | N20°01′48″ | Cattle farm | Armigeres | *Armigeres subalbatus* | 1 | 1 |
| 81 | 2023-HNWC-Ct | 2023.04.18-19 | WC | E110°35′06″ | N20°01′48″ | Cattle farm | Culex | *Culex tritaeniorhynchus* | 3 | 147 |
| 82 | 2023-HNWC-Ae | 2023.04.18-19 | WC | E110°35′06″ | N20°01′48″ | Cattle farm | Aedes | *Aedes albopictus* | 1 | 13 |
| 83 | 2023-HNQH-Mu | 2023.05.23-24 | QH | E110°13′51″ | N19°09′23″ | Cattle farm | Mansonia | *Mansonia uniformi*s | 1 | 30 |
| 84 | 2023-HNQH-CT | 2023.05.23-24 | QH | E110°13′51″ | N19°09′23″ | Cattle farm | Culex | *Culex tritaeniorhynchus* | 19 | 1650 |
| 85 | 2023-HNQH-As | 2023.05.23-24 | QH | E110°13′51″ | N19°09′23″ | Cattle farm | Armigeres | *Armigeres subalbatus* | 14 | 420 |
| 86 | 2023-HNHK-Ct | 2023.07.27 | HK | E110°32′17″ | N19°58′06″ | Cattle farm,Rice paddy | Culex | *Culex tritaeniorhynchus* | 22 | 1284 |
| 87 | 2023-HNHK-Cg | 2023.07.27 | HK | E110°32′17″ | N19°58′06″ | Cattle farm,Rice paddy | Culex | *Culex gelidus* | 2 | 30 |
| 88 | 2023-HNHK-黑 | 2023.07.27 | HK | E110°32′17″ | N19°58′06″ | Cattle farm,Rice paddy | Aedes | *Aedes cinereus* | 2 | 30 |
| 89 | 2023-HNHK-An | 2023.07.27 | HK | E110°32′17″ | N19°58′06″ | Cattle farm,Rice paddy | Anopheles | *Anopheles.sp* | 2 | 110 |
| 90 | 2023-HNHK-Al | 2023.07.27 | HK | E110°32′17″ | N19°58′06″ | Cattle farm,Rice paddy | Aedes | *Aedes lineatopennis* | 13 | 753 |
| 91 | 2023-HNHK-Av | 2023.07.27 | HK | E110°32′17″ | N19°58′06″ | Cattle farm,Rice paddy | Aedes | *Aedes vexans* | 8 | 465 |
| 92 | 2023-HNHK-Ae-male | 2023.07.27 | HK | E110°32′17″ | N19°58′06″ | Cattle farm,Rice paddy | Aedes | *Aedes albopictus* | 1 | 33 |
| 93 | 2023-HNHK-Ae | 2023.07.27 | HK | E110°32′17″ | N19°58′06″ | Cattle farm,Rice paddy | Aedes | *Aedes albopictus* | 2 | 107 |
| 94 | 2023-HNBT-Ct | 2023.05.25 | BT | E109°34′46″ | N18°41′28″ | Cattle farm | Culex | *Culex tritaeniorhynchus* | 4 | 330 |
| 95 | 2023-HNBT-As | 2023.05.25 | BT | E109°34′46″ | N18°41′28″ | Cattle farm | Armigeres | *Armigeres subalbatus* | 1 | 15 |
| 96 | 2023-HNBS-An | 2023.05.17 | BS | E109°26′28″ | N19°18′22″ | Cattle farm | Anopheles | *Anopheles.sp* | 1 | 22 |
| 97 | 2023-HNBS-As | 2023.05.17 | BS | E109°26′28″ | N19°18′22″ | Cattle farm | Armigeres | *Armigeres subalbatus* | 1 | 5 |
| 98 | 2023-HNBS-Ct | 2023.05.17 | BS | E109°26′28″ | N19°18′22″ | Cattle farm | Culex | *Culex tritaeniorhynchus* | 2 | 100 |
| 99 | 2022-HNLS-Cul | 2022.06.16-17 | LS | E110°02′24″ | N18°31′35″ | Peasant household | Culicoides | *Culicoides* | 2 | 700 |
| 100 | 2022-HNWN-Cul | 2022.04.25-27 | WN | E110°11′37″ | N18°41′32″ | Cattle farm | Culicoides | *Culicoides* | 8 | 2800 |
| 101 | 2023-HNQZ-Cul | 2023.05.18 | QZ | E109°42′37″ | N19°05′31″ | Cattle farm | Culicoides | *Culicoides* | 39 | 13650 |
| 102 | 2023-HNDZ-Cul | 2023.04.13-14 | DZ | E109°44′46″ | N19°25′52″ | Pigsty,Cattle farm | Culicoides | *Culicoides* | 1 | 350 |
| 103 | 2023-HNLS-Cul | 2023.07.24-25 | LS | E110°03′13″ | N18°37′03″ | Cattle farm | Culicoides | *Culicoides* | 1 | 350 |
| 104 | 2023-HNWN-Cul | 2023.08.02-04 | WN | E110°16′15″ | N18°48′08″ | Pigsty,Cattle farm | Culicoides | *Culicoides* | 6 | 2100 |
| 105 | 2023-HNQH-Cul | 2023.05.23-24 | QH | E110°13′51″ | N19°09′23″ | Cattle farm | Culicoides | *Culicoides* | 2 | 700 |
| 106 | 2023-HNBS-Cul | 2023.05.17 | BS | E109°26′28″ | N19°18′22″ | Cattle farm | Culicoides | *Culicoides* | 1 | 350 |

Note: * HK: Haikou city, WC: Wenchang city, QH: Qionghai city, WN: Wanning city, LS: Lingshui county, TC: Tunchang county, QZ: Qiongzhong county, BT: Baoting county, SY: Sanya city, DZ: Danzhou city, BS: Baisha county, CJ: Changjiang county, LD: Ledong county.
