# Supplementary material for: Detection and evolutionary characterization of arboviruses in mosquitoes and biting midges on Hainan Island, China, 2019–2023
Source: PLoS Negl Trop Dis. 2024 Oct 31;18(10):e0012642. doi: 10.1371/journal.pntd.0012642 (PMC11556698; doi:10.1371/journal.pntd.0012642)
Supplement: S3 Table — (DOCX) [file pntd.0012642.s003.docx]

S3 Table. Amino acid mutation analysis of JEV envelope protein (E) protein detected in Hainan Island

| Starin of JEV | Domain I | | |  | DomainⅡ | | | | | |  | DomainⅢ | | |  | Outside domain | | |
| --- | --- | --- | --- | --- | --- | --- | --- | --- | --- | --- | --- | --- | --- | --- | --- | --- | --- | --- |
|  | E138 | E176 | E177 |  | E107 | E129 | E222 | E244 | E264 | E279 |  | E315 | E327 | E366 |  | E439 | E447 |  |
| SA14-14-2  （AF495589.1） | K | V | A |  | F | T | A | G | H | M |  | V | S | A |  | R | G |  |
| SA14  （KU323483.1） | E | I | T |  | L | T | A | E | Q | K |  | A | S | A |  | K | G |  |
| HN-WN22-Cu-04 | E | I | T |  | L | M | S | E | Q | K |  | A | T | S |  | K | G |  |
| HN-WN22-Cu-14 | E | I | T |  | L | M | S | E | Q | K |  | A | T | S |  | K | G |  |
| **HN-WN22-Cu-18** | E | I | T |  | L | M | S | E | Q | K |  | A | T | S |  | K | G |  |
| **HN-QH23-Ct-01** | E | I | T |  | L | M | S | E | Q | K |  | A | T | S |  | K | G |  |
| **HN-QH23-Ct-13** | E | I | T |  | L | M | S | E | Q | K |  | A | T | S |  | K | G |  |
| HN-QH23-Ct-18 | E | I | T |  | L | M | S | E | Q | K |  | A | T | S |  | K | V |  |
| HN-QZ23-Ct-01 | E | I | T |  | L | M | S | E | Q | K |  | A | T | S |  | K | G |  |
| HN-BT23-Ct-03 | E | I | T |  | L | M | S | E | Q | K |  | A | T | S |  | K | G |  |
| HN-HK23-Ct-20 | E | I | T |  | L | M | S | E | Q | K |  | A | T | S |  | K | G |  |
