# Supplementary material for: Detection and evolutionary characterization of arboviruses in mosquitoes and biting midges on Hainan Island, China, 2019–2023
Source: PLoS Negl Trop Dis. 2024 Oct 31;18(10):e0012642. doi: 10.1371/journal.pntd.0012642 (PMC11556698; doi:10.1371/journal.pntd.0012642)
Supplement: S4 Table — (DOCX) [file pntd.0012642.s004.docx]

S4 Table. Amino acid mutation analysis of GETV envelope protein 2 protein detected in Hainan Island

| Starin of GETV | E109 | E134 | E205 | E269 | E283 | E323 | E368 | E374 | E378 |
| --- | --- | --- | --- | --- | --- | --- | --- | --- | --- |
| M1 | G | A | R | L | T | D | V | C | V |
| HNDZ1712-1 | D | A | S | V | I | E | A | G | I |
| HN-QH23-As-10 | D | T | S | V | T | E | A | G | I |
| HN-QH23-As-12 | D | T | S | V | T | E | A | G | I |
| HN-QH23-As-14 | D | T | S | V | T | E | A | G | I |
