# Supplementary material for: Detection and evolutionary characterization of arboviruses in mosquitoes and biting midges on Hainan Island, China, 2019–2023
Source: PLoS Negl Trop Dis. 2024 Oct 31;18(10):e0012642. doi: 10.1371/journal.pntd.0012642 (PMC11556698; doi:10.1371/journal.pntd.0012642)
Supplement: S5 Table — (DOCX) [file pntd.0012642.s005.docx]

S5A Table. Janpanese encephalities virus sequences information

| GenBank accession | Strain | Collection date | Location | Host | Genotype | Genotype |
| --- | --- | --- | --- | --- | --- | --- |
| KT957422.1 | M28 | 1977 | China: Yunnan | *Culex pseudovishnui* | GI | GI-a |
| MT253732.1 | TS00 | 2000 | Australia | Porcine | GI | GI-a |
| JF706282.1 | YN83-Meng83-54 | 1983 | China: Yunnan | Lasiohelea taiwana Shiraki | GI | GI-a |
| GQ902062.1 | 4790-85 | 1985 | Thailand | Homo sapiens | GI | GI-a |
| GQ902061.1 | B-1381-85 | 1985 | Thailand | *Pig* | GI | GI-a |
| MH184571.1 |  | 2017 | China: Guangdong | *Culex tritaeniorhynchus* | GI | GI-b |
| OM572546.1 | GX-059sd | 2017 | China: Guangxi | Mosquito | GI | GI-b |
| OM572538.1 | JN19-1 | 2019 | China: Shandong | Mosquito | GI | GI-b |
| OM572549.1 | ZJ18-66 | 2018 | China: Zhejiang | Mosquito | GI | GI-b |
| KX357114.1 | JS-1 | 2015 | China: Jiangsu | *Culex tritaeniorhynchus* | GI | GI-b |
| OM572535.1 | SDWS1607 | 2016 | China: Shandong | Mosquito | GI | GI-b |
| PP682372 | HN-WN22-Cu-18 | 2022 | China: Hainan | Culex.sp | GI | GI-b |
| PP682373 | HN-QH23-Ct-01 | 2023 | China: Hainan | *Culex tritaeniorhynchus* | GI | GI-b |
| PP682374 | HN-QH23-Ct-13 | 2023 | China: Hainan | *Culex tritaeniorhynchus* | GI | GI-b |
| MK495877.1 | NSBr/01/2017 | 2017 | South Korea | *Phoca largha* | GI | GI-b |
| OM572548.1 | LN1861 | 2018 | China: Liaoning | Mosquito | GI | GI-b |
| MT134112.1 | NX1889 | 2018 | China: Ningxia | Homo sapiens | GI | GI-b |
| OM572539.1 | GS1943 | 2019 | China: Gansu | Mosquito | GI | GI-b |
| OK423757.1 | JEV-SC-2020-1 | 2020 | China: Sichuan | Sus scrofa | GI | GI-b |
| OM572540.1 | SX19117 | 2019 | China: Shaanxi | Mosquito | GI | GI-b |
| JF706281.1 | YN05124 | 2005 | China: Yunnan | *Culex tritaeniorhynchus* | GI | GI-b |
| KY927815.1 | 639A37Cx-tri | 2014 | Cambodia | *Culex tritaeniorhynchus* | GI | GI-a |
| LC687612.1 | JEV-seal-UT1-2020 | 2021 | Japan | *Phoca vitulina* | GI | GI-b |
| PP336429.1 | VN1CT-03 | 2003 | Viet Nam | *Culex tritaeniorhynchus* | GI |  |
| MT253737.1 | Mab2496 | 1998 | Australia | Porcine | GII |  |
| HQ223285.1 | Bennett | 1951 | South Korea | Homo sapiens | GII |  |
| HQ223287.1 | JKT654 | 1978 | Indonesia | Mosquito | GII |  |
| JF706276.1 | HLJ02-134 | 2002 | China: Heilongjiang | *Culicoides sp* | GIII |  |
| MZ923735.1 | WH | 2014 | China | Pig | GIII |  |
| JN604986.1 | SA14-14-2 | 2002 |  | live-attenuated virus vaccine strain | GIII |  |
| AY184212.1 | JKT6468 | 1981 | Indonesia | Mosquito | GⅣ |  |
| LC579814.1 | 19CxBa-83-Cv | 2019 | Indonesia | *Culex vishnui* | GⅣ |  |
| HM596272.1 | Muar | 1952 | Malaysia | Homo sapiens | GⅤ |  |
| MT568538.1 | A18.3210 | 2018 | South Korea | *Culex bitaeniorhynchus* | GⅤ |  |
| AY037116.1 | TSV01 | 1994 | Australia | Patient |  |  |

S5B Table. Bluetongue virus sequences information

| GenBank accession | Strain | Collection date | Location | Host | Genotype |
| --- | --- | --- | --- | --- | --- |
| KC879616.1 | Y863 | 1979 | China: Yunnan | *Ovis aries* | BTV-01 |
| JN848760.1 | SZ97/1 | 1997 | China: Yunnan | *Ovis aries* | BTV-01 |
| KP821005.1 | GRE2001/01 | 2001 | Greece | *Ovis aries* | BTV-01 |
| KP339135.1 | BTV01IND2010-KRM07 | 2010 | India | *Ovis aries* | BTV-01 |
| MN698742.1 | V9820 | 2017 | Australia | *Bos taurus* | BTV-01 |
| KP696553.1 | BTV-1/IND2003/04 | 2003 | India |  | BTV-01 |
| KP821028.1 | ITL2002/02 | 2002 | Italy | *Culicoides obsoletus* | BTV-02 |
| OR603993.1 | BTV-3/NET2023 | 2023 | Netherlands | *Ovis aries* | BTV-03 |
| DQ191281.1 | 92069 | 2003 | Italy | *Culicoides imicola* | BTV-04 |
| KT945042.1 | V084 | 2012 | China: Yunnan | *Bos taurus* | BTV-05 |
| GQ506489.1 | P635 | 2010 | South Africa | *Culicoides* | BTV-06 |
| KT002579.1 | GDST008 | 2014 | China: Gangdong | *Bos taurus* | BTV-07 |
| LR797848.1 | V250.2 | 2018 | Switzerland | *Ovis aries* | BTV-08 |
| AB686223.1 |  | 2003 | Japan | *Culicoides brevitarsis* | BTV-09 |
| OP185805.1 | BTV-10/11 (4138) | 2011 | France | *Bos taurus* | BTV-10 |
| JQ972852.1 |  | 2010 | Germany | *Bos taurus* | BTV-11 |
| AB686216.1 |  | 1990 | Japan | *Culicoides* | BTV-12 |
| OP185795.1 | BTV-13/11 (4048) | 2011 | France | *Bos taurus* | BTV-13 |
| KP821096.1 | RUS2011/01 | 2011 | Russia | *Bos taurus* | BTV-14 |
| MH346492.1 | B105/YN/1996 | 1996 | China: Yunnan | *Bos taurus* | BTV-15 |
| AB686226.1 |  | 2008 | Japan | *Culicoides* | BTV-16 |
| KX599360.1 | BRA73 | 2014 | Brazil | *Ovis aries* | BTV-17 |
| MT078370.1 | Ref 18 | 2020 | South Africa | *Ovis aries* | BTV-18 |
| KP821075.1 | RSA1998/01 | 1998 | South Africa | *Capra hircus* | BTV-19 |
| MN710218.1 | RSArrrr/20 | 1975 | Australia | *Culicoides* | BTV-20 |
| MK250957.1 | YN/2017 | 2017 | China: Yunnan | *Capra hircus* | BTV-21 |
| MN710220.1 | RSArrrr/22 | 1992 | South Africa | *Culicoides* | BTV-22 |
| MT090656.1 | Ref 23 | 2020 | South Africa | *Ovis aries* | BTV-23 |
| KT945049.1 | V137 | 2012 | China: Yunnan | *Bos taurus* | BTV-24 |
| LR993250.1 | V301.4 | 2019 | Switzerland | *Capra hircus* | BTV-25 |
| HM590642.1 | KUW2010/02 | 2010 | Kuwait | *Ovis aries* | BTV-26 |
| KU760988.1 | BTV-27/FRA2014/v02 | 2014 | France | *Capra hircus* | BTV-27 |
| MN723881.1 | SPvvvv/03 | 2014 | Israel |  | BTV-28 |
| KX695171.1 | V196/XJ/2014 | 2014 | China: Xinjiang | *Capra hircus* | BTV-29 |
| PP682363 | HN-QZ23-Cul-12 | 2023 | China: Hainan | *Culicoides* |  |

S5C Table. Tembusu virus sequences information

| GenBank accession | Strain | Collection date | Location | Host | Cluster |
| --- | --- | --- | --- | --- | --- |
| MF621927.1 | DK/TH/CU-DTMUV2007 | 2007 | Thailand | Duck | Cluster 1 |
| KX097990.1 | D1921/1/3/MY | 2012 | Malaysia | Pekin duck | Cluster 1 |
| KR061333.1 | DK/TH/CU-1 | 2013 | Thailand | Duck | Cluster 2 |
| KY623437.1 | ZJ201505 | 2015 | China | Duck | Cluster 2 |
| JQ928189.1 | FJMH220 | 2010 | China: Fujian | Common shelduck | Cluster 2 |
| KX686576.1 | PY-2013 | 2013 | China | Layer duck | Cluster 2 |
| KF557893.1 | lq-1 | 2012 | China: Shandong | Wulong goose | Cluster 2 |
| MZ355579.1 | CTLN | 2020 | China | Egg-laying chicken | Cluster 3 |
| OM240640.1 | SD2021 | 2021 | China | Chicken | Cluster 3 |
| KT607936.1 | YN12193 | 2012 | China: Yunnan | Culex tritaeniorhynchus | Cluster 3 |
| KT607935.1 | YN12115 | 2012 | China: Yunnan | Culex tritaeniorhynchus | Cluster 3 |
| MH748542.1 | SD14 | 2014 | China: Shandong | Anas platyrhynchos | Cluster 3 |
| JX477685.2 | MM_1775 | 2012 | Malaysia | Mosuqito | Cluster 4 |
| JX477686.1 | Sitiawan virus | 2012 | Malaysia | Chicken | Cluster 4 |
| OP858811.1 | MLP46 | 2020 | China: Yunnan | Culex tritaeniorhynchus | Cluster 3 |
| OP858813.1 | FN249 | 2021 | China: Yunnan | Culex tritaeniorhynchus | Cluster 3 |
| OQ238827.1 | YN2020 | 2020 | China: Yunnan | Culex tritaeniorhynchus | Cluster 3 |
| PP682375 | HN-SY23-Ct-05 | 2023 | China: Hainan | Culex tritaeniorhynchus | Cluster 3 |
| PP682376 | HN-TC23-Cu-03 | 2023 | China: Hainan | Culex.sp | Cluster 3 |
| PP682377 | HN-WN23-Cg-02 | 2023 | China: Hainan | Culex gelidus | Cluster 3 |

S5D Table. Getah virus sequences information

| GenBank accession | Strain | Collection date | Location | Host | Group |
| --- | --- | --- | --- | --- | --- |
| MT121984.1 | AMM2021 | 1955 | Malaysia | Culex gelidus | G1 |
| MN849355.1 | MM2021 | 1955 | Malaysia | Culex gelidus | G1 |
| AF339484.1 | MM 2021 | 1955 | Malaysia | Culex gelidus | G1 |
| AB032553.1 | SAGV | 1956 | Japan | Mosquito | G2 |
| EU015061.1 | M1 | 1964 | China:Hainan | Culex sp | G3 |
| EF631998.1 | LEIV 16275 Mag | 2000 | Russia | Aedes sp | G4 |
| KY434327.1 | YN12031 | 2012 | China: Yunnan | Armigeres subalbatus | G4 |
| LC534253.1 | SW | 2017 | Thailand | Sus scrofa | G4 |
| ON828425.1 | HNDZ1712-1 | 2017 | China: Hainan | Culex tritaeniorhynchus | G3 |
| MZ736796.1 | GX1 | 2019 | China: Guangxi | Swine | G3 |
| MZ357112.1 | SCrph129 | 2020 | China: Sichuan | Ailurus fulgens | G3 |
| MZ357111.1 | SCrph328 | 2018 | China: Sichuan | Ailurus fulgens | G3 |
| PP682378 | HN-QH23-As-10 | 2023 | China: Hainan | Armigeres subalbatus | G3 |
| PP682379 | HN-QH23-As-12 | 2023 | China: Hainan | Armigeres subalbatus | G3 |
| PP682380 | HN-QH23-As-14 | 2023 | China: Hainan | Armigeres subalbatus | G3 |
| MW246769.1 | NM,JA_F2_18-8L-NH-Cxp-Y-1-1 | 2018 | China: Inner Mongolia | Culex pipiens | G3 |
| MZ736793.1 | HeB201707 | 2017 | China: Hebei | Sus scrofa | G3 |
| MZ388464.1 | GETV-XJ-2019-07 | 2017 | China: Xinjiang | horse | G3 |
| LC152056.1 | 12IH26 | 2012 | Japan | Culex tritaeniorhynchus | G3 |
| MH722255.1 | JL1707 | 2017 | China: Jilin | mosquito | G3 |
