# Supplementary material for: Detection and evolutionary characterization of arboviruses in mosquitoes and biting midges on Hainan Island, China, 2019–2023
Source: PLoS Negl Trop Dis. 2024 Oct 31;18(10):e0012642. doi: 10.1371/journal.pntd.0012642 (PMC11556698; doi:10.1371/journal.pntd.0012642)
Supplement: Attachment — Submitted filename: PNTD-D-24-00640 (1) (1).docx [file pntd.0012642.s006.docx]

**PLOS Neglected Tropical Diseases**

**Detection and evolutionary characterization of arboviruses in mosquitoes and biting midges on Hainan Island, China, 2019–2023**

--Manuscript Draft--

| **Manuscript Number:** | PNTD-D-24-00640 |
| --- | --- |
| **Full Title:** | Detection and evolutionary characterization of arboviruses in mosquitoes and biting midges on Hainan Island, China, 2019–2023 |
| **Short Title:** | Arbovirus in mosquitoes and biting midges, Hainan Island, China |
| **Article Type:** | Research Article |
| **Keywords:** | Arbovirus; Mosquitoes; Biting midges; Hainan Island |
| **Abstract:** | We conducted a large-scale survey of arboviruses in mosquitoes and biting midges to assess the presence and spread of mosquito-borne pathogens that are currently circulating on Hainan Island, China. RT-PCR assays were used to detect the arbovirus species, distribution, and infection rates in mosquitoes and biting midges. Cell inoculated and high throughput sequencing was performed to isolate viruses and assemble full viral genomes. Phylogenetic analysis was conducted to identify the viral genotypes and evolutionary relationships with known viruses. During 2019–2023, we collected 32,632 mosquitoes and 21,000 biting midges from 14 of 18 cities/counties on Hainan Island. Japanese encephalitis virus (JEV) was detected in mosquitoes from 5/18 cities/counties (28%), where the minimum infection rate (MIR) 1.57‰ (0.55‰- 2.60‰).Tembusu virus (TMUV) was detected inTunchang, Wanning and Sanya three cities/counties with MIR1.03‰ (0-2.20‰). Getah virus (GETV) was detected in Armigeres subalbatus collected from Qionghai city, where the MIR was 7.142‰ (0–15.23‰). Oya virus and Bluetongue virus (BTV)were detected in biting midges with MIRs of 0.42‰ (0–1.23‰) and 0.07‰ (0–10.22‰), respectively. Three JEV strains successfully isolated from Culex sp. and Culex tritaeniorhynchus and clustered together within the group of genotype I, which is the dominant genotype in China at present. Three TMUV strains were isolated for the first time on Hainan Island and belonged into Cluster 3. Three GETVs isolates from Ar. subalbatu were identified as Group 3. BTV was first time reported present in arthropod vector on Hainan Island, and the complete genome for one BTV strain was successfully assembled, which was classified as serotype 1 base on segment 2. |
| **Additional Information:** |  |
| **Question** | **Response** |
| **Government Employee**  Are you or any of the contributing authors an employee of the United States government?  Manuscripts authored by one or more US Government employees are not copyrighted, but are licensed under a [CC0](https://creativecommons.org/about/cc0) [Public Domain Dedication](https://creativecommons.org/about/cc0), which allows unlimited distribution and reuse of the article for any lawful purpose. This is a legal requirement for US Government employees. | No - No authors are employees of the U.S. government. |

*Powered by Editorial Manager® and ProduXion Manager® from Aries Systems Corporation*

| This will be typeset if the manuscript is accepted for publication. |  |
| --- | --- |
| **Financial Disclosure**  Enter a financial disclosure statement that describes the sources of funding for the work included in this submission. Review the [submission guidelines](http://journals.plos.org/plosntds/s/submission-guidelines#loc-financial-disclosure-statement) for detailed requirements. View published research articles from [*PLOS Neglected Tropical*](https://journals.plos.org/plosntds/) [*Diseases*](https://journals.plos.org/plosntds/) for specific examples.  This statement is required for submission and **will appear in the published article** if the submission is accepted. Please make sure it is accurate. | Yes |
| **Funded studies**  Enter a statement with the following details:   - Initials of the authors who received each award - Grant numbers awarded to each author - The full name of each funder - URL of each funder website - Did the sponsors or funders play any role in the study design, data collection and analysis, decision to publish, or preparation of the manuscript? |  |
| Did you receive funding for this work? |  |
| Please add funding details.  as follow-up to "**Financial Disclosure**  Enter a financial disclosure statement that describes the sources of funding for the work included in this submission. Review the [submission guidelines](http://journals.plos.org/plosntds/s/submission-guidelines#loc-financial-disclosure-statement) for detailed requirements. View published research articles from [*PLOS Neglected Tropical*](https://journals.plos.org/plosntds/) [*Diseases*](https://journals.plos.org/plosntds/) for specific examples.  This statement is required for submission and **will appear in the published article** if the submission is accepted. Please make sure it is accurate. | 1.National Key Research and Development Program of China (2022YFC2302700) 2.the National Natural Science Foundation of China (U22A20363)   3. the Hainan Province Science and Technology Special Fund (ZDYF2023SHFZ134, ZDKJ2021035) 4. the Youth Program of Wuhan Institute of Virology (2023QNTJ-03) |

*Powered by Editorial Manager® and ProduXion Manager® from Aries Systems Corporation*

| **Funded studies**  Enter a statement with the following details:   - Initials of the authors who received each award - Grant numbers awarded to each author - The full name of each funder - URL of each funder website - Did the sponsors or funders play any role in the study design, data collection and analysis, decision to publish, or preparation of the manuscript? |  |
| --- | --- |
| Did you receive funding for this work?" |  |
| Please select the country of your main research funder (please select carefully as in some cases this is used in fee calculation).  as follow-up to "**Financial Disclosure**  Enter a financial disclosure statement that describes the sources of funding for the work included in this submission. Review the [submission guidelines](http://journals.plos.org/plosntds/s/submission-guidelines#loc-financial-disclosure-statement) for detailed requirements. View published research articles from [*PLOS Neglected Tropical*](https://journals.plos.org/plosntds/) [*Diseases*](https://journals.plos.org/plosntds/) for specific examples.  This statement is required for submission and **will appear in the published article** if the submission is accepted. Please make sure it is accurate. | CHINA - CN |
| **Funded studies**  Enter a statement with the following details:   - Initials of the authors who received each award - Grant numbers awarded to each author - The full name of each funder - URL of each funder website - Did the sponsors or funders play any role in the study design, data collection and analysis, decision to publish, or preparation of the manuscript? |  |
| Did you receive funding for this work?" |  |
| **Competing Interests** | The authors declare that they have no competing interests. |

*Powered by Editorial Manager® and ProduXion Manager® from Aries Systems Corporation*

| On behalf of all authors, disclose any competing interests that could be perceived to bias this work.  This statement will be typeset if the manuscript is accepted for publication.  Review the instructions link below and PLOS NTDs’ [competing interests](https://journals.plos.org/plosntds/s/competing-interests#loc-what-represents-a-competing-interest) policy to determine what information must be disclosed at submission. |  |
| --- | --- |
| **Data Availability**  Provide a **Data Availability Statement** in the box below. This statement should detail where the data used in this submission can be accessed. This statement will be typeset if the manuscript is accepted for publication.  Before publication, authors are required to make all data underlying their findings fully available, without restriction. Review our [PLOS Data Policy](https://journals.plos.org/plosntds/s/data-availability) page for detailed information on this policy. Instructions for writing your Data Availability statement can be accessed via the Instructions link below. | All the data are open access. |

*Powered by Editorial Manager® and ProduXion Manager® from Aries Systems Corporation*

Manuscript	[Click here to access/download;Manuscript;Manuscript.pdf](https://www2.cloud.editorialmanager.com/pntd/download.aspx?id=1318738&guid=40f6c019-4c25-4cc7-a8d6-6ae827fd85fa&scheme=1)

# 1 Detection and evolutionary characterization of arboviruses in mosquitoes and

2 **biting midges on Hainan Island, China, 2019–2023**
3 **Authors:** Qun Wu1,2,3#, Dingwei Sun3#, Wahid Zaman^1,2^, Fei Wang^1,2^, Doudou
4 Huang^1^, Haixia Ma^1^, Shunlong Wang^1,2^, Ying Liu^3^, Puyu Liu^3^, Xuexia Zeng^3^,
5 Zhiming Yuan^1,2*^, Han Xia^1,2,3*^

# 6 Affiliations:

7 ^1^Wuhan Institute of Virology, Chinese Academy of Sciences, Wuhan, Hubei, China
8 ^2^University of Chinese Academy of Sciences, Beijing, China
9 ^3^Hainan Provincial Center for Disease Control and Prevention, Haikou, Hainan,
10 China
11 ^4^Hubei Jiangxia Laboratory, Wuhan, Hubei, China
12 #These two authors contributed equally.
13 ^*^Correspondence: Zhiming Yuan, yzm@ wh. iov. cn; Han Xia, hanxia@ wh. iov. cn

# 14 Abstract

15 We conducted a large-scale survey of arboviruses in mosquitoes and biting midges to
16 assess the presence and spread of mosquito-borne pathogens that are currently
17 circulating on Hainan Island, China. RT-PCR assays were used to detect the arbovirus
18 species, distribution, and infection rates in mosquitoes and biting midges. Cell
19 inoculation and high throughput sequencing was performed to isolate the viruses and
20 assemble full viral genomes. Phylogenetic analysis was conducted to identify the viral
21 genotypes and evolutionary relationships with known viruses. During 2019–2023,
22 32,632 mosquitoes and 21,000 biting midges were collected from 14 of 18
23 cities/counties on Hainan Island. Japanese encephalitis virus (JEV) was detected in
24 *Culex* mosquitoes from 5/14 cities/counties, where the minimum infection rate (MIR)
25 was 1.57(0.552.60) per 1,000 females tested. Tembusu virus (TMUV) was detected in *Culex*
26 mosquitoes from 3/14 cities/counties with MIR1.03‰ (0-2.20‰). Getah virus
27 (GETV) was detected in *Armigeres* mosquitoes from 1/14 cities/counties with MIR
28 7.142‰ (0–15.23‰). Oya virus and Bluetongue virus (BTV) were detected in biting
29 Midges, with MIRs of 0.42‰ (0–1.23‰) and 0.07‰ (0–10.22‰), respectively. Three
30 JEV strains were isolated and clustered together within the group of genotype
31 I, which is currently the dominant genotype in China. Three TMUV strains were
32 isolated for the first time on Hainan Island and belonged into Cluster 3. Three isolated
33 GETVs were identified as Group 3. BTV was reported for the first time on Hainan
34 Island, and the complete genome for one BTV strain was successfully assembled,
35 which was classified as serotype 1 based on the sequences of segment 2.

# 36 Author Summary

37 Large-scale surveillance of arboviruses in mosquitoes was performed 40 years ago on
38 Hainan Island. Recently, only limited studies have been conducted in certain regions
39 of Hainan. We conducted comprehensive screening of arboviruses in
40 mosquitoes and biting midges on Hainan Island during 2019–2023. Except the
41 arbovirus such as JEV, GTEV, and OYAV, which has been reported circulating on
42 Hainan Island, TMUV and BTV were detected for the first time during the survey.
43 These findings highlight the increasing risk of arbovirus transmission in Hainan
44 Island. It is crucial to prioritize extensive and continuous surveillance of arboviruses
45 carried by mosquitoes and biting midges in local regions in order to help prevent the
46 spread of emerging and re-emerging arboviral diseases.

# 47 Introduction

48 The World Health Organization reported that vector-borne diseases account for more
49 than 17% of all infectious diseases worldwide and cause more than 700,000 deaths
50 annually. More than 3.9 billion people in over 129 countries are at risk of contracting
51 dengue fever, with an estimated 96 million symptomatic cases and 40,000 new cases
52 every year [1]. At least 530 viruses are listed in the U.S. Centers for Disease Control
53 and Prevention arbovirus catalog (https://wwwn.cdc.gov/arbocat/), and around 134
54 are known to cause human disease [2].
55 Mosquitoes and midges are important vectors
56 of arboviruses that infect humans or animals . *Aedes* mosquitoes are major vector of
57 diseases caused by dengue, chikungunya, yellow fever, and Zika viruses. In
58 addition, *Culex* mosquitoes transmit Japanese encephalitis and West
59 Nile viruses [3]. More than 50 viruses have been isolated from *Culicoides* spp.
60 worldwide. Most of the viruses belong to the families *Sedoreoviridae*. (e.g., African
61 horse sickness virus (AHSV) and bluetongue virus (BTV)), *Rhabdoviridae* (e.g.,
62 bovine ephemeral fever virus) and *Peribunyaviridae* (e.g., Akabane virus,
63 Schmallenberg virus, or Oropouche virus). Among them, AHSV and BTV cause sever
64 animal diseases, which have been classified as Category A animal disease by World
65 Organization for Animal Health [4-6].
66 Hainan Island, located in the tropical and subtropical zones, is separated from mainland
67 China by the Qiongzhou Strait, and it comprises a unique and independent natural
68 ecosystem with a typical tropical marine monsoon climate. These conditions are
69 conducive to the survival and reproduction of various arthropod vectors, including
70 mosquitoes, ticks, and biting midges, that facilitate the circulation of arboviruses
71 among vectors and vertebrates [7, 8]. Hainan Island is an endemic region for dengue,
72 with outbreaks in 1979, 1980–1991, and 2019, and for Japanese encephalitis
73 with outbreaks during 1952–1990 [9-11]. Arbovirus surveillance
74 conducted among vectors on Hainan Island during 1960 to1990s
75 detected and isolated dengue (DENV), Japanese encephalitis
76 (JEV), chikungunya , and Ross river viruses from mosquitoes, ticks, and bats
77 [12]. However, rapid economic development as well as increased tourism and
78 urbanization have significantly altered the ecological environment on Hainan
79 Island and the distribution of vectors. Despite these changes, no large-scale systematic
80 investigations of arboviruses in mosquitoes and biting midges have been conducted
81 on the island since the 1990s [13]. In the present study, we assessed the
82 arboviruses circulating in mosquitoes and biting midges on Hainan Island based on a
83 large-scale field survey.

# 84 Methods

85 **Mosquito sampling**
86 Using lamp t or animal tent trapping techniques, mosquitoes were collected
87 from March to October, and biting midges were captured from April to August.
88 Mosquitoes and biting midges were collected from 14 out of 18 cities/counties on
89 Hainan Island between 2019 and 2023 (Fig. 1), covering the island’s east, west, south,
90 north, and central regions. Female adult mosquitoes were identified to species and pooled
91 (30–60 mosquitoes/pool) by collection site, date, and species. We identified
92 biting midges only to genus level (*Culicoides*), with 300–400 midges/pool.

# 93 Sample preparation and RNA extraction

94 Mosquitoes and biting midges were assigned to different pools according to the
95 collection site, species, and sex. Next, 1000 μL of Roswell Park Memorial Institute
96 medium was added to each pool, before triturated with the
97 cryogenic grinding method by using a High-Speed Low-Temperature Tissue Grinding
98 Machine (Servicebio, China Wuhan) with two 30-s cycles at 50 Hz. After sufficient
99 grinding**,** macerated mosquitoes and biting midges were centrifuged at 15,000 × *g*
100 (4°C for 30 min) to remove any cell debris and bacteria [14]. Supernatants were
101 stored at −80°Ce. RNA was extracted from 200 μL of each
102 homogenized supernatant sample with 600 μL TRIzol® LS Reagent (Cat No:
103 10296010, Thermo Fisher, USA) and a Direct-zolTM RNA MiniPrep kit (Cat No:
104 R2050, Zymo Research, USA) according to the manufacturer’s instructions.

# 105 Arboviruses detection through RT-PCR

106 Mosquito or biting midge extracts were screened for arboviruses by RT-PCR according to previously described
107 methods [13, 15, 16]. Briefly, complementary DNA (cDNA) was synthesized using a
108 TransScript One-Step gDNA Removal and cDNA Synthesis SuperMix Kit (Cat No:
109 AH311, TransGen Biotech, China). The reaction conditions were 95°C for 2 min,
110 followed by 34 cycles at 95°C for 15 s, 52°C for 15 s, and 72°C for 15 s; and then
111 72°C for 5 min, before reducing the temperature to 4°C until the product was removed
112 from the thermocycler. The primer sequences and characteristics are shown in S1
113 Table. The minimum infection rate (MIR) was calculated as: (number of positive
114 pools/total number of individual mosquitoes or midges tested) × 1000, and 95%
115 confidence intervals were determined using the chi-square calculator in SPSS version
116 21 (International Business Machines Corporation).

# 117 Arboviruses isolation and purification

118 Viruses were isolated as described in a previous study [14]. Briefly, the remaining
119 parts of the mosquito or midge homogenates were removed from storage at –80°C and
120 kept on ice. Microfiltered supernatants (100 μL) from each pool were inoculated
121 into each well of 24-well plates containing C6/36 (*Aedes albopictus* RNAi-deficient)
122 cells as passage one. After 1 h of incubation at 28°C (C6/36), the inoculum was
123 removed and replaced with RPMI (with 2% fetal bovine serum (FBS) and 2%
124 (penicillin-streptomycin-amphotericin B (PSA) Solution, and the cell plates were
125 placed into an incubator at 28°C with 5% CO 2 for 5–7 days, followed by two passages
126 in C6/36 cells. The remaining mosquito or midge homogenates also were inoculated
127 into BHK-21(baby hamster kidney) cells according to the same steps described above,
128 but with an incubation temperature of 37°C and using cell maintenance fluid
129 comprising Dulbecco’s modified Eagle medium with 2% FBS and 2% PSA. The
130 supernatants (200 μL) from the third passage in C6/36 cells were seeded onto BHK-
131 21 cell plates (24-well plates) and incubated for 5–7 days to monitor cytopathic
132 effects.

# 133 Sequencing and phylogenetic analysis

134 Envelope genes in JEV fragments were amplified from cDNA with positive pools.
135 The primers were as follows [17]: JEV-E-F, 5′-TGYTGGTCGCTCCGGCTTA-3′;
136 and JEV-E-R, 5′-GATGTCAATGGCACATCCAGT-3′. The amplicons were purified
137 with a Gel Extraction Kit (Cat No: D2500-02, Omega, USA) and each cDNA was
138 cloned into the pCE3 Blunt Vector (Cat No: C603-1, Vazyme, China) and
139 transformed into DH5α Competent Cells (Cat No: B528413, Sangon Biotech, China).
140 Sequencing libraries were prepared with a VAHTS®Universal V8 RNA-seq Library
141 Prep Kit for Illumina (Cat No: NR605, Vazyme, China) according to the
142 manufacturer’s instructions. Whole genomes were sequenced by next-generation
143 sequencing, and phylogenetic analyses were performed as described previously [14].
144 The sequences were identified by BLAST searches using GenBank. The sequences
145 obtained were aligned with MegAlign and neighbor-joining trees were generated with
146 MEGA v. 11 using 1000 bootstrap replicates. Phylogenetic trees were visualized,
147 modified, and annotated by using Tree Visualization by One Table (tvBOT;
148 htt[ps://www.chiplot.onl](http://www.chiplot.online/normalTree.html))i[ne/normalTree.html)](http://www.chiplot.online/normalTree.html)) [18].

# 149 Results

150 **Mosquito and biting midge samples**
151 During 2019 to 2023, we collected 32,632 mosquitoes from 14
152 cities/counties on Hainan Island identified as 11 species,
153 comprising *Culex tritaeniorhynchus* (34.96%), *Aedes albopictus* (16.51%), *Armigeres*
154 *subalbatus* (9.78%), *Culex gelidus* (5.31%), *Aedes lineatopennis* (2.31%), *Anopheles*
155 sp. (2.14%), *Aedes vexans* (1.42%), *Mansonia uniformis* (0.45%), *Culex*
156 *quinquefasciatus* (0.25%), *Aedes cinereus* (0.09%), *Culex bitaeniorhynchus* (0.06%),
157 and unidentified *Culex* sp (26.69%) (Fig. 1 and S2 Table). Approximately 21,000
158 *Culicoides* biting midges were collected from six cities/counties (Qiongzhong,
159 Danzhou, Lingshui, Wanning, Qionghai, and Baisha) (Fig. 1 and S2 Table).

# 160 Fig. 1. Sampling locations for mosquitoes and *Culicoides* biting midges between

161 **2019 and 2023 on Hainan Island, China.** HK: Haikou city, WC: Wenchang city,
162 QH: Qionghai city, WN: Wanning city, LS: Lingshui county, TC: Tunchang county,
163 QZ: Qiongzhong county, BT: Baoting county, SY: Sanya city, DZ: Danzhou city, BS:
164 Baisha county, CJ: Changjiang county, LD: Ledong county.

# 165 Detection of arboviruses in mosquitoes and biting midges

166 Fifteen mosquito pools and two biting midge pools tested positive by RT-PCR,
167 comprising nine mosquito pools positive for JEV, three for Tembusu virus (TMUV) and three
168 for Getah virus (GETV), and one biting midge pool positive for Oya virus (OYAV) and
169 one for Bluetongue virus (BTV) (Table 1). In particular, JEV was detected in *Culex*
170 sp. from Wanning on the eastern coast of Hainan Island in 2022 and *Cx.*
171 *tritaeniorhynchus* from four cities/counties (Haikou, Qionghai, Qiongzhong,
172 and Baoting) during 2023 covering the northern, eastern coastal, central, and
173 southeastern regions of Hainan Island, respectively. The collection specific MIRs ranged from 0.78
174 (0–2.31) to 4.55(0–13.50) per 1,000. In addition, TMUV was detected in *Cx. gelidus* in
175 Wanning, *Cx. tritaeniorhynchus* in Sanya, and *Culex* sp. in Tunchang during 2023,

178	with MIRs per 1,000 of 1.40 (0–4.15), 0.69 (0–2.05), and 1.35 (0–3.99‰),

179 respectively. Furthermore, GETV was detected in *Ar. subalbatus* from Qionghai with
180 an MIR of 7.14(0–15.23‰) (Table 1). OYAV was detected in biting midges from
181 Waning in 2022 with an MIR of 0.42‰ (0–1.23‰), and BTV was detected in biting
182 midges from Qiongzhong in 2023 with an MIR of 0.07‰ (0–10.22‰).

# 183 Virus isolation and phylogenetic analyses

184 Based on the nucleotide sequence of the whole genome, JEV is currently classified
185 into five different genotypes: GI, GII, GIII, GIV, and GV (Fig. 2A) [19]. Three strains
186 of JEV were successfully isolated. One strain (HN-WN22-Cu-18) was isolated from
187 *Culex* sp*.* in Wanning during 2022, and the other two (HN-QH23-Ct-01 and HN-
188 QH23-Ct-03) were from *Cx. tritaeniorhynchus* in Qionghai during 2023. The latter 2 isolates
189 clustered together within GI (Fig. 2A), which is the dominant genotype in China at
190 present. Phylogenetic analysis based on the whole-genome of JEV showed that HN-
191 WN22-Cu-18 and HN-QH23-Ct-01 clustered with two strains identified in mosquito
192 samples collected during 2017 from Guangxi and Guangdong provinces in China, whereas
193 HN-QH23-Ct-13 was more closely related to the samples obtained from *Cx.*
194 *tritaeniorhynchus* collected during 2015 in Jiangsu province in China (Fig. 2A).
195 **Table 1 Arboviruses identified in mosquitoes and biting midges on Hainan Island, 2019–2023 PCR Screening**

**Virus	Strain	Location	Year	Mosquito/ biting midge**

**sample genus/ species**

**Habitant**

**Positive**

**No. pools**

**Positive pool rate**

**NO.**

**individual**

**MIR (‰) 95%CI**

**Isolation	Accession No.**

4.55

NO	NA

|  | | | | | **Pools** |  | **(%)** | **s** |
| --- | --- | --- | --- | --- | --- | --- | --- | --- |
| WN22-Cu-18  WN22-Cu-04 | WN | 2022 | *Culex.sp* | Cattle farm | 3 | 24 | 12.50 | 2242 1.34 YES PP682372  （0-2.85） NO NA |
| WN22-Cu14 |  |  |  |  |  |  |  | NO NA |
| QH23-Ct-01  Janpanese QH23-Ct-13  encephalitie | QH | 2023 | *Cx. tritaeniorhynchus* | Cattle farm | 3 | 19 | 15.79 | 1650 1.82 YES PP682373  （0-3.88） YES PP682374 |
| s QH23-Ct-18  virus HK23-Ct-20 | HK | 2023 | *Cx. tritaeniorhynchus* | Cattle farm Rice paddy | 1 | 22 | 4.55 | NO NA  1284 0.78 NO NA  （0-2.31） |

| (JEV) | QZ23-Ct-01 | QZ | 2023 | *Cx. tritaeniorhynchus* | Cattle farm | 1 | 4 | 25.00 | 220 |
| --- | --- | --- | --- | --- | --- | --- | --- | --- | --- |
|  | BT23-Ct-03 | BT | 2023 | *Cx. tritaeniorhynchus* | Cattle farm | 1 | 4 | 25.00 | 330 |
|  | SY23-Ct-05 | SY | 2023 | *Cx. tritaeniorhynchus* | Cattle farm | 1 | 21 | 4.76 | 1448 |

（0-13.50）

Tembusu virus (TMUV)

TC23-Cu-03	TC	2023	*Culex.sp*	Rubber Grove

,Cattle

3.03

（0-8.99）

0.69

（0-2.05）

1	8	12.50	742	（ 1.35

0-3.99）

NO	NA

YES	PP682375

YES	PP682376

WN23-Cg-02	WN	2023	*Cx. gelidus*	Cattle farm	1	12	8.33	714	1.40

0-4.15

（	）

QH23-As-10

0-15.23）

YES	PP682377

YES	PP682378

Getah virus (GETV),

QH23-As-12	QH	2023	*Ar. subalbatus*	Cattle farm	3	14	21.43	420	（ 7.14

YES	PP682379

QH23-As-14	YES	PP682380

(0-1.23)

Oya virus (OYAV)

Bluetongue

WN22-Cul-04	WN	2022	*Culicoides. sp*	Cattle farm	1	8	12.50	2400	0.42

0.07

NO	NA

PP682362;PP68 2363;PP682364

;PP682365;PP6

virus (BTV)

QZ23-Cul-12	QZ	2023	*Culicoides. sp*	Cattle farm	1	39	2.56	13650

(0-0.22)	NO

82366;PP68236

7;PP682368;PP

682369;PP6823

70; PP682371

| 196 | Note: HK: Haikou city, WC: Wenchang city, QH: Qionghai city, WN: Wanning city, LS: Lingshui county, TC: Tunchang county, QZ: Qiongzhong county, BT: Baoting county, SY: Sanya city, DZ: Da |
| --- | --- |
| 197 | nzhou city, BS: Baisha county, CJ: Changjiang county, LD: Ledong county. |
| 198 |  |

199 The E protein of JEV is considered the main site for host–virus attachment and it
200 consists of three structural domains: domain I (DI; E1–E51, E137–E196, and E293–
201 E311), domain II (DII; E52–E137 and E197–E292), and domain III (DIII; E310–
202 E411) [21-23]. The deduced amino acid differences in E protein sequences were
203 aligned for comparison among the newly sequenced strains, strains detected in
204 mosquitoes from Hainan Island, SA14 strain (KU323483.1), and the vaccine strains
205 (SA14-14-2 and AF495589.1) currently used in China (S3 Table). Fourteen amino
206 acid residues in the newly detected Hainan JEV strains differed from those in the live
207 attenuated vaccine SA14-14-2-derived strain: three in DI with E138(Lys→Glu/Gln),
208 E176(Val→le), and E177(Ala →Thr); six in DII with E107(Phe→Leu),
209 E129(Thr→Met), E222(Ala→Ser), E244(Gly→Glu/Gln), E264(His→Gln), and
210 E279(Met→Lys); three in DIII with E315(Val→Ala), E327(Ser→Thr), and
211 E366(Ala→Ser); and two outside the domains with E439(Arg→Lys) and
212 E447(Gly→Val). Five mutation sites differed between the newly detected Hainan
213 JEV strains and the SA14 strain comprising: E129(Thr→Met), E222(Ala→Ser),
214 E327(Ser→Thr), E366(Ala→Ser), and E447(Gly→Val).
215 We did not successfully obtain the BTV isolate, but the complete genome of BTV
216 HN-QZ23-Cul-12 obtained from biting midges in Qiongzhong during 2023 was
217 successfully assembled. Phylogenetic analysis based on segment 2 (VP2) showed that
218 this Hainan BTV strain belonged to serotype 1 and it was closely related to BTVs
219 isolated from India and Greece (Fig. 2B) with similarities of 95.66% and 94.50%,
220 respectively.
221 Three strains of TMUV (HN-TC23-Cu-03, HN-WN23-Cg-02, and HN-SY23-Ct-05)
222 were isolated for the first time in Hainan (Table 1) and assigned to Cluster 3.They
223 were closely related to strains identified in mosquito samples collected during 2012
224 and 2020 in Yunnan (Fig. 2C).
225 In addition, three GETV isolates (HN-QH23-As-10, HN-QH23-As-12, and HN-
226 QH23-As-14) isolated from *Ar. subalbatus* during 2023 in Qionghai were identified
227 as G3, and they clustered into a separate clade with the closest evolutionary distance
228 to strain HND1712-1 isolated from *Cx. tritaeniorhynchus* during 2017 in Hainan (Fig.
229 2D). The deduced amino acid sequences of envelope protein 2 were aligned to
230 compare the newly isolated GETV strains (HN-QH23-As-10, HN-QH23-As-12, and
231 HN-QH23-As-14) with the M1 strain and HND1712-1 strain isolated previously in
232 Hainan. Nine mutation sites differed between the GETV strains (HN-QH23-As-10,
233 HN-QH23-As-12, and HN-QH23-As-14) and the M1 strain (S4 Table), i.e.,
234 E109(Gly→Asp), E134(Ala→Thr), E205(Arg→Ser), E269(Leu→Val),
235 E323(Asp→Glu/Gln), E368(Val→Ala), E374(C→Gly), and E378(V→I). Two
236 mutation sites comprising E134(Ala→Thr) and E283(Le→Thr) differed compared
237 with the HND1712-1 strain. Thus, the GETV strains present in the Hainan
238 area were quite different from the M1 strain isolated in 1964, but no additional frequent
239 mutations had appeared in recent years.

# 240 Fig. 2. Phylogenetic analysis of newly identified arboviruses isolated from

241 **mosquitoes/midges with complete genomes in Hainan Island during 2019–2023.**
242 (A) Phylogenetic tree constructed based on the complete genome of Japanese
243 encephalitis virus (JEV). (B) Phylogenetic tree constructed based on the complete
244 sequence of segment 2 in Bluetongue virus (BTV). (C) Phylogenetic tree constructed
245 based on the complete genome of Tembusu virus (TMUV). (D) Phylogenetic tree
246 constructed based on the complete genome of Getah virus (GETV). The blue circle
247 indicates the viral strain reported previously in Hainan Island, and red highlights
248 indicate the viral strains obtained in the present study. Sequences information in S5
249 Table.

# 250 Discussion

251 To strengthen epidemiological investigations of arboviruses and meet the need
252 for early disease warning, RT-PCR detection, virus isolation, and deep

sequencing were used in combination in our study

253 JEV was isolated previously during 1983–1985 in Hainan [24], but there were no full
254 genome sequence information and there were no further detections of JEV in mosquitoes from
255 Hainan until 30 years later in 2017 [13]. The detection of JEV in *Cx.*
256 *tritaeniorhynchus* and *Culex* sp. pools from five cities and counties in Hainan
257 confirmed that this species was the main vector of JEV and that it was widespread in
258 Hainan. The phylogenetic tree showed that all strains belonged to GI in the same
259 manner as those tested in 2017–2018 [13], and they were closely related to strains
260 collected during 2015 and 2017 from Jiangsu, Guangxi, and Guangdong provinces in
261 China (Fig. 2A). The JEV isolates from mosquitoes, pigs, birds, and human belonged
262 to G3 before 2008, and GI was only isolated subsequently from mosquitoes [25].
263 However, in the last 20 years, JEV GI has replaced GIII as the dominant genotype in
264 China [20, 26]. Our results indicated that JEV GI is also the dominant genotype in
265 Hainan. Further investigations are needed to identify whether other JEV genotypes
266 might be present in Hainan Province.
267 The E protein is the crucial virulence determinant for JEV [27] and eight substitutions
268 (E107, E138, E176, E177, E264, E279, E315, and E439) in the E protein gene affect
269 the critical amino acids related to virus attenuation, and thus they are often used as the
270 critical targets for quality control in vaccine strains [22, 28]. In our study, the newly
271 detected Hainan JEV strains differed completely from the vaccine strains (SA14-14-2)
272 in terms of the eight substitutions (S3 Table), but they were the same as the those in
273 the SA14 strain, thereby indicating that the JEV virulence has not changed
274 dramatically in Hainan. Another five amino acid residues also differed
275 (E129Thr→Met, E222Ala→Ser, E327Ser→Thr, E366Ala→Ser, and E447Gly→Val),
276 and some or all were detected in previous studies in Shanghai province (E222, E327,
277 and E366) and Shandong province (E129, E222, E327, and E366) [29-31]. In addition
278 to the critical amino acid mutations, other mutations such as those at E129, E222,
279 E327, E366, and E447 might cause immune escape from the vaccine, but further
280 research is needed.
281 The first BTV isolate in China was found during 1979 in Yunnan province [32], and
282 BTV infections in cattle were investigated subsequently in 23 Chinese
283 provinces [33]. There have been no previous reports of BTV detection or isolation on
284 Hainan Island. In the present study, strain HN-QZ23-Cul-12 was detected and it's full
285 genome was assembled for the first time from biting midges collected on Hainan
286 Island and identified as serotype 1 (Fig. 2B). Midges (*Culicoides* spp.) play important
287 roles in the transmission of BTV [34]. BTV can be transmitted and circulate
288 continuously between cattle and midges, and thus cattle at the Qiongzhong collection
289 site may have been infected with BTV, which highlights the need for increased
290 monitoring of infections in local animals.
291 TMUV is an emerging mosquito-borne flavivirus and it was previously identified in
292 mosquitoes in Shandong [35] and Yunnan [36] provinces in China. Phylogenetic
293 analyses of the whole genomes showed that TMUVs could be divided into four
294 clusters [37]. Cluster 1, Cluster 2, and Cluster 4 mainly contain strains isolated from
295 ducks, geese and chickens, and contribute to the prevalence of disease, e.g.,
296 strain D1921/1/3/MY caused outbreaks of neurological disease characterized by
297 progressive paralysis in broiler duck flocks aged between 4 and 7 weeks old in some
298 areas of Malaysia [38]. Cluster 3 is considered a novel cluster of TMUVs, which
299 contains strains originating from mosquitoes that have acquired attenuated
300 pathogenicity compared with those in other clusters, especially Cluster 2 [39]. There
301 have been no previous reports of TMUV isolates from mosquitoes collected on
302 Hainan Island. The three Hainan TMUV strains were grouped within
303 Cluster 3 based on the whole-genome tree, and these were closely related to strains
304 identified in mosquitoes and chickens (Fig. 2C). The results showed that the TMUV
305 strains from Hainan were similar to other TMUV strains that originated in mosquitoes
306 and chickens and that mosquitoes may have played a significant role in the spread
307 of TMUV among local chickens in Hainan. A previous study found surprisingly high
308 rates of seropositivity and Duck Tembusu virus (DTMUV) positivity by
309 RT-PCR in duck farm workers in China. Some people who had no contact with
310 ducks also had high neutralizing antibody titers to DTMUV in Thailand [40, 41].
311 Thus, it is important to strengthen TMUV screening in mosquitoes and chickens, but
312 special attention should also be paid to health monitoring for personnel involved in
313 poultry farming in Hainan.
314 GETV is a neglected mosquito-borne Alphavirus that causes pyrexia, body rash, and
315 leg edema in horses, and fetal death and reproductive disorders in pigs [42]. GETVs
316 have been isolated from 17 different mosquito species belonging to five genera in
317 Culicidae (*Culex*, *Anopheles*, *Armigeres*, *Aedes*, and *Mansonia)*, as well as from
318 midges in Eurasia, and have been found in 65% (22/34) of the provinces in China
319 by March 2022 [43]. The isolation of GETVs on Hainan Island was expected because

GETV was isolated in this area in 1964 [44] and

320 about 50 years later in 2017–2018 [13]. Phylogenetic analyses of whole genomes
321 showed that the newly isolated Hainan GETV strains (HN-QH23-As-10, HN-QH23-
322 As-12, and HN-QH23-As-14) were distant from the M1 strains isolated previously in
323 1964 but close to the HND1712-1 strain isolated during 2017 in Hainan (Fig. 2D),
324 presumably because the HND1712-1, HN-QH23-As-10, HN-QH23-As-12, and HN-
325 QH23-As-14 strains and M1 strains had different origins. The newly isolated Hainan GETV strains
326 were assigned to G3 in the whole-genome tree (Fig. 2D). GETV G3 is the main group
327 that causes animal diseases, and these viruses have caused multiple outbreaks
328 in horses and pigs, anddisease in cattle and blue foxes in recent years [42, 43,

339	45].

340 Large-scale surveillance of arboviruses in mosquitoes on Hainan Island was
341 performed 40 years ago. However, only limited studies have been conducted recently
342 in certain regions of Hainan. Thus, in the present study, we comprehensively screened
343 for arboviruses in mosquitoes and biting midges on Hainan Island during 2019–2023.
344 Human cases of JE have rarely been reported on Hainan Island in recent years due to
345 JE vaccination programs. Our results showed that JEV is still circulating in
346 mosquitoes on Hainan Island and the genotype of these new isolates was determined
347 as GI, which is consistent with those found on the mainland. All of the new GETV
348 strains were isolated from *Ar. subalbatus* mosquitoes, which indicates that other
349 mosquito species can carry GETV in addition to *Culex* mosquitoes. Importantly,
350 outbreaks caused by TMUV in poultry and BTV in livestock have been reported on
351 mainland China, but our detection of TMUV and BTV in vectors for the first time
352 indicates a concerning rise in the diversity of arboviruses and the risk of infection on
353 Hainan Island. These findings demand increased awareness and proactive measures to
354 safeguard community health. DENV was not found in this study, possibly due to the
355 limited number of samples collected, where most of the *Ae. albopictus* samples were
356 collected from Sanya and Qiongzhong, with lower numbers collected in other areas.
357 Furthermore, recent dengue outbreaks in Hainan were primarily linked to imported
358 cases, and thus there may be a low prevalence of DENVs carried by local *Aedes*
359 mosquitoes on Hainan Island at present.

# 360 Supporting information

361 S1 Table. Primers used in heminested RT-PCR or RT-PCR assays for detecting
362 arboviruses. (DOCX)
363 S2 Table. Geographic and species information for samples collected on Hainan Island

364	during 2019–2023. (DOCX)

365 S3 Table. Amino acid mutation analysis for JEV envelope protein (E) protein detected
366 on Hainan Island. (DOCX)
367 S4 Table. Amino acid mutation analysis for GETV envelope protein 2 protein
368 detected on Hainan Island. (DOCX)
369 S5 Table. Sequences information in Fig 2. (DOCX)

# 370 Acknowledgments

371 This study was supported by the National Key Research and Development Program of
372 China (2022YFC2302700), National Natural Science Foundation of China
373 (U22A20363), Hainan Province Science and Technology Special Fund
374 (ZDYF2023SHFZ134 and ZDKJ2021035), and Youth Program of Wuhan Institute of
375 Virology (2023QNTJ-03).The authors would like to thank the Haikou, Wenchang,
376 Qionghai, Wanning, Lingshui, Sanya, Tunchang, Qiongzhong, Wuzhishan, Baoting,
377 Danzhou,Baisha,Changjiang and Ledong Centers for Disease Control and Prevention,
378 who provided help with mosquitoes and biting midge sample collection. We thank the
379 assistance provided by Institutional Center for Shared Technologies and Facilities of
380 Wuhan Institute of Virology, CAS.

# 381 Author Contributions

382 **Conceptualization:** Han Xia and Zhiming Yuan.
383 **Data curation:** Qun Wu, Dingwei Sun, Fei Wang, Shunlong Wang.
384 **Formal Analysis:** Han Xia, Zhiming Yuan, Fei Wang.
385 **Funding Acquisition:** Han Xia and Zhiming Yuan.
386 **Investigation:** Qun Wu, Dingwei Sun, Ying Liu, Puyu Liu, Xuexia Zeng.
387 **Methodology:** Qun Wu, Dingwei Sun, Han Xia.
388 **Project Administration:** Han Xia and Zhiming Yuan.
389 **Resources:** Qun Wu, Dingwei Sun, Ying Liu, Puyu Liu, Xuexia Zeng.
390 **Supervision:** Han Xia and Zhiming Yuan.
391 **Validation:** Han Xia and Zhiming Yuan.
392 **Visualization:** Qun Wu, Han Xia, Dingwei Sun, Wahid Zaman.
393 **Writing – Original Draft Preparation:** Qun Wu, Han Xia, Dingwei Sun.
394 **Writing – Review & Editing:** Qun Wu, Dingwei Sun, Wahid Zaman, Fei Wang,
395 Shunlong Wang, Ying Liu, Puyu Liu, Xuexia Zeng, Zhiming Yuan, Han Xia.
396 References
397 1.	WHO. Vector-borne diseases 2020 [updated 2020. Available from:
398 [https://www.who.int/news-room/fact-sheets/detail/vector-borne-diseases.](https://www.who.int/news-room/fact-sheets/detail/vector-borne-diseases)
399 2.	Gubler DJ. Human Arbovirus Infections Worldwide. Annals of the New York
400 Academy of Sciences. 2010;951(WEST NILE VIRUS: DETECTION):13-24.
401 3.	Liu QY. [Epidemic profile of vector-borne diseases and vector control strategies in the
402 new era]. Chinese Journal of Vector Biology and Control. 2019;30(1):1-6+11. Chinese
403 4.	Mellor PS, Boorman J, Baylis M. Culicoides biting midges: their role as arbovirus
404 vectors. Annual review of entomology. 2000;45:307-40. 405	[https://doi.org/10.1146/annurev.ento.45.1.307.](https://doi.org/10.1146/annurev.ento.45.1.307)

406	5.	Carpenter S, Veronesi E, Mullens B, Venter G. Vector competence of Culicoides for

407	arboviruses: three major periods of research, their influence on current studies and

408	future directions. Revue scientifique et technique (International Office of Epizootics). 409	2015;34(1):97-112. [https://doi.org/10.20506/rst.34.1.2347.](https://doi.org/10.20506/rst.34.1.2347)

410	6.	Sick F, Beer M, Kampen H, Wernike K. Culicoides Biting Midges-Underestimated

411	Vectors for Arboviruses of Public Health and Veterinary Importance. Viruses. 412	2019;11(4). <https://doi.org/10.3390/v11040376>.

413	7.	Li SP, Jiang F, Lu H, Kang X, Wang YH, Zou Z, et al. Mosquito Diversity and

414	Population Genetic Structure of Six Mosquito Species From Hainan Island. Frontiers in 415	genetics. 2020;11:602863. <https://doi.org/10.3389/fgene.2020.602863>.

416	8.	Li YJ, Zhou GF, Zhong SF, Wang XM, Zhong DB, Hemming-Schroeder E, et al.

417	Spatial heterogeneity and temporal dynamics of mosquito population density and

418	community structure in Hainan Island, China. Parasites & vectors. 2020;13(1):444. 419	[https://doi.org/10.1186/s13071-020-04326-5.](https://doi.org/10.1186/s13071-020-04326-5)

420 9.	Fan WF, Yu SR, Cosgriff TM. The reemergence of dengue in China. Reviews of
421 infectious diseases. 1989;11 Suppl 4:S847-53.
422 [https://doi.org/10.1093/clinids/11.supplement_4.s847.](https://doi.org/10.1093/clinids/11.supplement_4.s847)
423 10.	Du J, Zhang LY, Hu XY, Peng RY, Wang GY, Huang Y, et al. Phylogenetic Analysis
424 of the Dengue Virus Strains Causing the 2019 Dengue Fever Outbreak in Hainan, 425	China. Virologica Sinica. 2021;36(4):636-43. [https://doi.org/10.1007/s12250-020-](https://doi.org/10.1007/s12250-020-00335-x) 426	[00335-x.](https://doi.org/10.1007/s12250-020-00335-x)

427	11.	Yan XJ, Sun LY, Zeng XX. [Results of epidemic encephatitis B case monitoring

428	system in Hainan in 2008 ]. China Tropical Medicine. 2009;9(10):2027-8. Chinese 429

430	12.	Zheng YY, Wang ZY, Liang GD. [Review on arbovirus studies in Hainan province,

431	China] Chines Journal of Vector Biology and Control. 2011;22(6):607-10. Chinese

432	13.	Fan N, Sun DW, Cheng R, Fu SH, Zeng LH, Wu Q, et al. [Isolation and identification

433	of Arbovirus in Hainan province, 2017-2018]. Zhonghua liuxingbingxue zazhi.

434	2020;41(2):236-43. [https://doi.org/10.3760/cma.j.issn.0254-6450.2020.02.018.](https://doi.org/10.3760/cma.j.issn.0254-6450.2020.02.018) Chinese

435 14.	Ren NJ, Wang XY, Liang MY, Tian S, Ochieng C, Zhao L, et al. Characterization of a
436 novel reassortment Tibet orbivirus isolated from Culicoides spp. in Yunnan, PR China.
437 The Journal of general virology. 2021;102(9). [https://doi.org/10.1099/jgv.0.001645.](https://doi.org/10.1099/jgv.0.001645)
438 15.	Xue ZJ, Zhao N, Wang J, Song XP, Meng FX, Liang WQ, et al. Establishment and
439 Application of Heminested RT-PCR Assay for Detection of Mosquito-Borne Flavivirus
440 — Guizhou Province, China, 2018. China CDC Weekly. 2021;3:4-9. 441	[https://doi.org/10.46234/ccdcw2021.001.](https://doi.org/10.46234/ccdcw2021.001)

442	16.	Xue ZJ, Zhao N, Wang J, Song XP, Meng FX, Liang WQ, et al. [Establishment and

443	application of RT-hemi-nested PCR assay for detection of mosquito-borne

444	alphaviruses] Chines Journal of Vector Biology and Control. 2021;32(2):132-8. 445	<https://doi.org/DOI>: 10.11853/j.issn.1003.8280.2021.02.003. Chinese

446	17.	LV SY, WANG JL, LI N, HU Q, HE YW, ZHAO DH, et al. [Nucleotide Detection of

447	Mosquito Japanese Encephalitis Virus and Sequence Analysis of PrM and E Genes in

448	Upper Pearl River Region]. China Animal Husbandry & Veterinary Medicine.

449	2017;44(2):336-43. <https://doi.org/10.16431/j.cnki.1671-7236.2017.02.005>. Chinese

450 18.	Xie JM, Chen YR, Cai GJ, Cai RL, Hu Z, Wang H. Tree Visualization By One Table
451 (tvBOT): a web application for visualizing, modifying and annotating phylogenetic
452 trees. Nucleic acids research. 2023;51(W1):W587-w92.
453 [https://doi.org/10.1093/nar/gkad359.](https://doi.org/10.1093/nar/gkad359)
454 19.	Yu X, Cheng G. Adaptive Evolution as a Driving Force of the Emergence and Re-
455 Emergence of Mosquito-Borne Viral Diseases. Viruses. 2022;14(2). 456	[https://doi.org/10.3390/v14020435.](https://doi.org/10.3390/v14020435)

457	20.	Li F, Feng Y, Wang GW, Zhang WJ, Fu SH, Wang ZS, et al. Tracing the

458	spatiotemporal phylodynamics of Japanese encephalitis virus genotype I throughout

459	Asia and the western Pacific. PLoS neglected tropical diseases. 2023;17(4):e0011192.

460	<https://doi.org/10.1371/journal.pntd.0011192>.

461	21.	Roy U. Structural and molecular analyses of functional epitopes and escape mutants in

462	Japanese encephalitis virus envelope protein domain III. Immunologic research. 463	2020;68(2):81-9. [https://doi.org/10.1007/s12026-020-09130-y.](https://doi.org/10.1007/s12026-020-09130-y)

464	22.	YAN RT, TANG DY, ZHANG S, HAN CY, YANG ZG, LUO L, et al. [Research

465	progress on the virulence-related amino acid sites of Japanese encephalitis virus E

466	protein]. Chinese Veterinary Science. 2021;51(7):908-12. 467	[https://doi.org/10.16656/j.issn.1673-4696.2021.0094.](https://doi.org/10.16656/j.issn.1673-4696.2021.0094)

468	23.	Luca VC, AbiMansour J, Nelson CA, Fremont DH. Crystal structure of the Japanese

469	encephalitis virus envelope protein. Journal of virology. 2012;86(4):2337-46. 470	[https://doi.org/10.1128/jvi.06072-11.](https://doi.org/10.1128/jvi.06072-11)

471 24.	Wenzhou C, Yuben C, Yue L, Jishen K, Mingjie C, Chuangqing C, et al. [Two strains
472 of Japanese encephalitis virus isolated from *Culex tritaeniorhynchus* for the first time
473 in Hainan Island]. South China Journal of Preventive Medicine. 1986;02:30-4. Chinese
474 25.	Wang H, Liang G. Epidemiology of Japanese encephalitis: past, present, and future
475 prospects. Therapeutics and clinical risk management. 2015;11:435-48. 476	[https://doi.org/10.2147/tcrm.S51168.](https://doi.org/10.2147/tcrm.S51168)

477	26.	Zhang W, Yin Q, Wang H, Liang G. The reemerging and outbreak of genotypes 4 and

478	5 of Japanese encephalitis virus. Frontiers in cellular and infection microbiology. 479	2023;13:1292693. [https://doi.org/10.3389/fcimb.2023.1292693.](https://doi.org/10.3389/fcimb.2023.1292693)

480	27.	Leng SL, Huang R, Feng YN, Peng LJ, Yang J, Li YH. The pre membrane and

481	envelope protein is the crucial virulence determinant of Japanese encephalitis virus.

482	Microbial pathogenesis. 2020;148:104492.

483	<https://doi.org/10.1016/j.micpath.2020.104492>.

484	28.	Yu Y. Phenotypic and genotypic characteristics of Japanese encephalitis attenuated live

485	vaccine virus SA14-14-2 and their stabilities. Vaccine. 2010;28(21):3635-41. 486	<https://doi.org/10.1016/j.vaccine.2010.02.105>.

487	29.	Deng X, Yan JY, He HQ, Yan R, Sun Y, Tang XW, et al. Serological and molecular

488	epidemiology of Japanese Encephalitis in Zhejiang, China, 2015-2018. PLoS neglected 489	tropical diseases. 2020;14(8):e0008574. [https://doi.org/10.1371/journal.pntd.0008574.](https://doi.org/10.1371/journal.pntd.0008574)

490	30.	Tao Z, Liu G, Wang M, Wang H, Lin X, Song L, et al. Molecular epidemiology of

491	Japanese encephalitis virus in mosquitoes during an outbreak in China, 2013. Scientific 492	reports. 2014;4:4908. [https://doi.org/10.1038/srep04908.](https://doi.org/10.1038/srep04908)

493	31.	Fang Y, Zhang Y, Zhou ZB, Xia S, Shi WQ, Xue JB, et al. New strains of Japanese

494	encephalitis virus circulating in Shanghai, China after a ten-year hiatus in local

495	mosquito surveillance. Parasites & vectors. 2019;12(1):22. 496	[https://doi.org/10.1186/s13071-018-3267-9.](https://doi.org/10.1186/s13071-018-3267-9)

497	32.	Zhu J, Yang H, Li H, Xiao L, Wang J, Li N, et al. Full-Genome Sequence of

498	Bluetongue Virus Serotype 1 (BTV-1) Strain Y863, the First BTV-1 Isolate of Eastern

499	Origin Found in China. Genome announcements. 2013;1(4). 500	[https://doi.org/10.1128/genomeA.00403-13.](https://doi.org/10.1128/genomeA.00403-13)

501	33.	Gong QL, Wang Q, Yang XY, Li DL, Zhao B, Ge GY, et al. Seroprevalence and Risk

502	Factors of the Bluetongue Virus in Cattle in China From 1988 to 2019: A

503	Comprehensive Literature Review and Meta-Analysis. Frontiers in veterinary science. 504	2020;7:550381. [https://doi.org/10.3389/fvets.2020.550381.](https://doi.org/10.3389/fvets.2020.550381)

505	34.	Li N, Meng J, He Y, Wang W, Wang J. Potential roles of Culicoides spp. (Culicoides

506	imicola, Culicoides oxystoma) as biological vectors of bluetongue virus in Yuanyang

507	of Yunnan, P. R. China. Frontiers in cellular and infection microbiology. 508	2023;13:1283216. [https://doi.org/10.3389/fcimb.2023.1283216.](https://doi.org/10.3389/fcimb.2023.1283216)

509	35.	Tang Y, Diao Y, Chen H, Ou Q, Liu X, Gao X, et al. Isolation and genetic

510	characterization of a tembusu virus strain isolated from mosquitoes in Shandong,

511	China. Transboundary and emerging diseases. 2015;62(2):209-16. 512	[https://doi.org/10.1111/tbed.12111.](https://doi.org/10.1111/tbed.12111)

513	36.	Hu D, Wu C, Wang R, Yao X, Nie K, Lv Q, et al. Persistence of Tembusu Virus in

514	Culex tritaeniorhynchus in Yunnan Province, China. Pathogens (Basel, Switzerland). 515	2023;12(3). [https://doi.org/10.3390/pathogens12030490.](https://doi.org/10.3390/pathogens12030490)

516	37.	Liao JY, Tang H, Xiong WJ, Liu TN, Li JY, Xia JY, et al. Isolation and

517	characterization of a novel goose ovaritis-associated cluster 3 Tembusu virus. Poultry 518	science. 2023;102(9):102867. [https://doi.org/10.1016/j.psj.2023.102867.](https://doi.org/10.1016/j.psj.2023.102867)

519	38.	Homonnay ZG, Kovács EW, Bányai K, Albert M, Fehér E, Mató T, et al. Tembusu -like

520	flavivirus (Perak virus) as the cause of neurological disease outbreaks in young Pekin

521	ducks. Avian pathology : journal of the WVPA. 2014;43(6):552-60. 522	[https://doi.org/10.1080/03079457.2014.973832.](https://doi.org/10.1080/03079457.2014.973832)

523	39.	Fang Y, Hang T, Yang LM, Xue JB, Fujita R, Feng XS, et al. Long-distance spread of

524	Tembusu virus, and its dispersal in local mosquitoes and domestic poultry in

525	Chongming Island, China. Infectious diseases of poverty. 2023;12(1):52. 526	[https://doi.org/10.1186/s40249-023-01098-9.](https://doi.org/10.1186/s40249-023-01098-9)

527	40.	Tang Y, Gao X, Diao Y, Feng Q, Chen H, Liu X, et al. Tembusu virus in human,

528	China. Transboundary and emerging diseases. 2013;60(3):193-6. 529	[https://doi.org/10.1111/tbed.12085.](https://doi.org/10.1111/tbed.12085)

530	41.	Pulmanausahakul R, Ketsuwan K, Jaimipuk T, Smith DR, Auewarakul P, Songserm T.

531	Detection of antibodies to duck tembusu virus in human population with or without the

532	history of contact with ducks. Transboundary and emerging diseases. 2022;69(2):870- 533	3. [https://doi.org/10.1111/tbed.13998.](https://doi.org/10.1111/tbed.13998)

534	42.	Liu H, Zhang X, Li LX, Shi N, Sun XT, Liu Q, et al. First isolation and

535	characterization of Getah virus from cattle in northeastern China. BMC veterinary 536	research. 2019;15(1):320. [https://doi.org/10.1186/s12917-019-2061-z.](https://doi.org/10.1186/s12917-019-2061-z)

537	43.	Li B, Wang H, Liang GD. Getah Virus (Alphavirus): An Emerging, Spreading

538	Zoonotic Virus. Pathogens (Basel, Switzerland). 2022;11(8). 539	[https://doi.org/10.3390/pathogens11080945.](https://doi.org/10.3390/pathogens11080945)

540 44.	Li XD, Qiu FX, Yang H, Rao YN, Calisher CH. Isolation of Getah virus from
541 mosquitos collected on Hainan Island, China, and results of a serosurvey. The
542 Southeast Asian journal of tropical medicine and public health. 1992;23(4):730-4.
543 45.	Shi N, Li LX, Lu RG, Yan XJ, Liu H. Highly Pathogenic Swine Getah Virus in Blue
544 Foxes, Eastern China, 2017. Emerging infectious diseases. 2019;25(6):1252-4. 545	[https://doi.org/10.3201/eid2506.181983.](https://doi.org/10.3201/eid2506.181983)

546

Figure	[Click here to access/download;Figure;Fig 1.tif](https://www2.cloud.editorialmanager.com/pntd/download.aspx?id=1318735&guid=f99598ed-d6ed-48f8-90f7-5b3f352f4e7b&scheme=1)

Figure	[Click here to access/download;Figure;Fig 2.tif](https://www2.cloud.editorialmanager.com/pntd/download.aspx?id=1318736&guid=f278d55f-cc58-47ec-8f56-4bebb078ed30&scheme=1)

Table	[Click here to access/download;Table;Table1.docx](https://www2.cloud.editorialmanager.com/pntd/download.aspx?id=1318737&guid=cbd1fe45-49e2-4727-8de8-0d30db668e2f&scheme=1)

**Table 1.	Arboviruses identified from mosquitos and biting midges in Hainan Island, 2019-2023**

**PCR Screening**

**Virus	Strain	Location	Year	Mosquito/ biting midge**

**sample genus/ species**

**No.**

**Habitant**

**Positive**

**No.**

**Positive pool rate**

**NO.**

**individual**

**MIR (‰) 95%CI**

**Isolation	Accession**

4.55

（	NO	NA

|  | | | | | **Pools** | **pools** | **(%)** | **s** |
| --- | --- | --- | --- | --- | --- | --- | --- | --- |
| WN22-Cu-18  WN22-Cu-04 | WN | 2022 | *Culex.sp* | Cattle farm | 3 | 24 | 12.50 | 2242 1.34 YES PP682372  （0-2.85） NO NA |
| WN22-Cu14 |  |  |  |  |  |  |  | NO NA |
| QH23-Ct-01  Janpanese QH23-Ct-13  encephaliti | QH | 2023 | *Cx. tritaeniorhynchus* | Cattle farm | 3 | 19 | 15.79 | 1650 1.82 YES PP682373  （0-3.88） YES PP682374 |
| es QH23-Ct-18  virus HK23-Ct-20 | HK | 2023 | *Cx. tritaeniorhynchus* | Cattle farm Rice paddy | 1 | 22 | 4.55 | NO NA  1284 （ 0.78 ） NO NA  0-2.31 |

| (JEV) | QZ23-Ct-01 | QZ | 2023 | *Cx. tritaeniorhynchus* | Cattle farm | 1 | 4 | 25.00 | 220 |
| --- | --- | --- | --- | --- | --- | --- | --- | --- | --- |
|  | BT23-Ct-03 | BT | 2023 | *Cx. tritaeniorhynchus* | Cattle farm | 1 | 4 | 25.00 | 330 |
|  | SY23-Ct-05 | SY | 2023 | *Cx. tritaeniorhynchus* | Cattle farm | 1 | 21 | 4.76 | 1448 |

0-13.50）

Tembusu virus (TMUV)

Getah virus (GETV),

TC23-Cu-03

WN23-Cg-02

QH23-As-10 QH23-As-12

3.03

（0-8.99）

0.69

（0-2.05）

1.35

| TC | 2023 | *Culex.sp* | Rubber Grove  ,Cattle | 1 | 8 | 12.50 | 742 |
| --- | --- | --- | --- | --- | --- | --- | --- |
| WN | 2023 | *Cx. gelidus* | Cattle farm | 1 | 12 | 8.33 | 714 |
| QH | 2023 | *Ar. subalbatus* | Cattle farm | 3 | 14 | 21.43 | 420 |
| WN | 2022 | *Culicoides. sp* | Cattle farm | 1 | 8 | 12.50 | 2400 |

（0-3.99）

1.40

（0-4.15）

7.14

（0-15.23）

NO	NA

YES	PP682375

YES	PP682376

YES	PP682377

YES	PP682378

YES	PP682379

QH23-As-14	YES	PP682380

Oya virus (OYAV)

Bluetongue

WN22-Cul- 04

0.42

(0-1.23)

0.07

NO	NA

PP682362; PP682363; PP682364; PP682365;

virus (BTV)

QZ23-Cul-12	QZ	2023	*Culicoides. sp*	Cattle farm	1	39	2.56	13650

(0-0.22)	NO

PP682366; PP682367; PP682368; PP682369; PP682370;

PP682371

Note: HK: Haikou city, WC: Wenchang city, QH: Qionghai city, WN: Wanning city , LS: Lingshui county, TC: Tunchang county, QZ: Qiongzhong county, BT: Baoting county, SY: Sanya c ity, DZ: Danzhou city, BS: Baisha county, CJ: Changjiang county, LD: Ledong county.

Supporting Information - Compressed/ZIP File Archive

[Click here to access/download](https://www2.cloud.editorialmanager.com/pntd/download.aspx?id=1318739&guid=1d75ebdb-6f82-4efb-b524-7657568e136f&scheme=1)

[**Supporting Information - Compressed/ZIP File Archive** S1-S5 Table.zip](https://www2.cloud.editorialmanager.com/pntd/download.aspx?id=1318739&guid=1d75ebdb-6f82-4efb-b524-7657568e136f&scheme=1)
