# Supplementary material for: Detection and evolutionary characterization of arboviruses in mosquitoes and biting midges on Hainan Island, China, 2019–2023
Source: PLoS Negl Trop Dis. 2024 Oct 31;18(10):e0012642. doi: 10.1371/journal.pntd.0012642 (PMC11556698; doi:10.1371/journal.pntd.0012642)
Supplement: Attachment — Submitted filename: Response to Editor and Reviewer-0929.docx [file pntd.0012642.s007.docx]

**Cover Letter in Response to Reviewer’s Comments**

Dear Editor and Reviewers,

Thank you for recognizing our work. We have fully revised the manuscript according to the editor and reviewer’s comments. Response to each comment is listed below. For your convenience, we have marked the text of the response in blue and the changes to the manuscript in red.

Thanks for your consideration.

Sincerely,

Han Xia, Ph.D

Professor

Key Laboratory of Virology and Biosafety

Wuhan Institute of Virology, Chinese Academy of Sciences

Wuhan, 430207, China

Tel: 86-027-87998215

E-mail: hanxia@wh.iov.cn

**Editor:**

**COMMENT 1:** Line 25 and many other places in the manuscript. Why report rates to two decimals of a percent? If one less or one more positive mosquito had been detected, the MIR would have changed by more than a full percent for many of the locations. This is reporting data to a level of accuracy not supported by the data and makes the reader question the accuracy of all of the data. Don’t report rates to more than one decimal place. See line 28 where if one less had been found, the MIR would have been 0, while if one more had been found, it would have been 14%, so a MIR of 7.142 is really meaningless.

**RESPONSE 1:** We thank the editor for the critical comments and helpful suggestions.

(1) The minimum infection rate (MIR) was calculated as (number of positive pools/total number of individual mosquitoes or midges tested) × 1000.

(2) We have updated all the MIRs in the manuscript to keep only one decimal place.

**COMMENT 2:** Line 51: Was it 40,000 new cases or 40,000 deaths? I believe that there were about 96 million new cases.

**RESPONSE 2:** Thanks for pointing out this. It has been corrected as “40,000 deaths”.

**COMMENT 3:** Lines 185-187: You stated this correctly. Yes, you detected JEV RNA in 9 pools of mosquitoes, but only detected actual virus in three pools.

**RESPONSE 3:** Yes, JEV was detected positive in nine pools by RT-PCR targeting the E gene, but only three JEV strains were isolated from three of the nine pools.

**COMMENT 4:** Line 195 (Table 1): As indicated in comment 3, you only detected JEV in 3 of the 9 JEV RNA detections. If JEV was not detected, isn’t it likely that only a small piece of noninfectious RNA was present in these other six pools? Should these unconfirmed positives but used to calculate a MIR? Similar comment for line 215 and BTV.

**RESPONSE 4:** Thanks for your comment.

(1) JEV was detected in nine pools by RT-PCR. The full E gene was successfully amplified from six pools, which did not successfully acquire the JEV isolate. The amplification band for the E gene from these pools was strong and specific, confirming that JEV was there (SFigure list below).

**SFigure. Detection of E gene of JEV infection in mosquito by RT-PCR.** Lane 1 to 6 was the pool of HN-WN22-Cu04, HN-WN22-Cu14, HN-QH23-Ct-18, HN-BT23-Ct03, HN-QZ23-Ct01, and HN-HK23-Ct20.

(2) Calculate an MIR based on RT-PCR results is common since the virus isolation from the field sample is not easy. For example, Fang Y, et al. [1] and MacIntyre C, et al. [2] calculate an MIR based on RT-PCR results in their research.

Ref：

[1] Fang Y, Hang T, Yang LM, et al. Long-distance spread of Tembusu virus, and its dispersal in local mosquitoes and domestic poultry in Chongming Island, China. Infect Dis Poverty. 2023 May 22;12(1):52. doi: 10.1186/s40249-023-01098-9. PMID: 37218001; PMCID: PMC10200701.

[2] MacIntyre C, Guarido MM, Riddin MA, et al. Survey of West Nile and Banzi Viruses in Mosquitoes, South Africa, 2011-2018. Emerg Infect Dis. 2023 Jan;29(1):164-169. doi: 10.3201/eid2901.220036. PMID: 36573591; PMCID: PMC9796198.

**COMMENT 5:** Lines 347-349: Why include the sentence on JE in the middle of the discussion of GETV? More importantly, the mere detection of GETV from a mosquito species does not mean that the species is a competent vector and that it is able to carry (i.e., transmit) that virus. However, I am fairly certain that it is a competent vector.

**RESPONSE 5:**

(1) Starting with line 361 is the concluding paragraph; the sentence on JE was not in the middle of the discussion of GETV.
(2) We agree that the mere detection of GETV from a mosquito species does not mean that the species is a competent vector and can carry (i.e., transmit) that virus. We have added this statement to the discussion in lines 362-364

**COMMENT 6:** Lines 355-356: Was it the limited number of samples collected or was it the limited number of Ae. aegypti and Ae. albopictus collected?

**RESPONSE 6:** It has been corrected as “the limited number of *Ae. aegypti* and *Ae. albopictus* collected”. (Page 21, lines 375-376)

**COMMENT 7:** Line 396+: References: These need to be formatted properly.

a. Only the first word and proper nouns in a reference title should be capitalized. See references 2, 6, 7, and many others.

b. The first letter of each word in a journal title should be capitalized. See references 4, 14, 18, 20, and many others.

c. Is it the “Chinese Journal of Vector Biology and Control” as in reference 3 or the “Chine Journal of Vector Biology and Control” as in reference 12?

d. Shouldn’t scientific names be in italics? See reference 14

e. Proper nouns, such as “Tembusu” should be capitalized. See reference 35 and others.

**RESPONSE 7:** We have corrected these errors according to your suggestions. Page 24-28, lines 423-589).

**COMMENT 8:** Line 66: Minor, but “…located in…zones” is a non-essential clause and should be separated by commas, i.e., “Island, located in…zones, is separated…”

**RESPONSE 8:** The commas have been added in line 70.

**COMMENT 9:** Line 76: only proper nouns are capitalized in a virus name, so this should be chikungunya virus. Note, it was done correctly on line 57.

**RESPONSE 9:** It has been corrected, and we have checked the whole manuscript for the writing of the virus name.

**COMMENT 10:** Line 124: What is RPMI? I know that it is Roswell Park Memorial Institute medium, but RPMI should have been established on line 95.

**RESPONSE 10:** The RPMI abbreviation has been established on lines 107-108.

**COMMENT 11:** Line 125: There should be a space between “(PSA)” and “solution,” and why capitalize “solution?” See also line 128 where there needs to be a space between “-21” and “baby…”

**RESPONSE 11:** The space has been added accordingly.

**COMMENT 12:** Line 162 (Fig. 1): It might be nice to indicate that the clear areas were not sampled and even include their names.

**RESPONSE 12**: Fig.1 has been updated as per your suggestion.

**COMMENT 13:** Line 171: “Bluetongue” should be bluetongue, but more importantly, BTV was established on line 61.

**RESPONSE 13**: It has been corrected.

**COMMENT 14**: Lines 251-253: This sentence is a bit confusing. Should, “…not only the RT-PCR detection, virus isolation, and deep sequencing were used in combination in this study.” have been, “…not only the RT-PCR detection, but virus isolation and deep sequencing were used in combination with RT-PCR in this study.”

**RESPONSE 14**: Thanks. It has been corrected as “…not only the RT-PCR detection, but virus isolation and deep sequencing were used in combination with RT-PCR in this study.” (Page17, lines 284-285).

**COMMENT 15**: Line 258: Shouldn’t “…isolate, but the…” be “…isolate, the full…” without “but?”

**RESPONSE 15**: It has been removed from this sentence.

**COMMENT16**: Lines 261-262: Shouldn’t “…mosquitoes Hainan…” be “…mosquitoes from Hainan…?”

**RESPONSE 16**: It has been corrected as “…mosquitoes from Hainan…” (Page 17, line 287).

**COMMENT17:** Lines 269-270: Is it “G3” as on line 269 or “GIII” as on line 270?

**RESPONSE 17:** It has been corrected as “GIII” (Page 17, line 295).

**COMMENT 18:** Line 279: “…as the those…” should be “as those…”

**RESPONSE 18:** It has been corrected as “as those” (Page18, line 305).

**COMMENT 19:** Line 280: Should “attenuation” be changed to “virulence?”

**RESPONSE 19:** It has been corrected as “virulence” (Page 18, line 306).

**COMMENT 20:** Line 309: Should “In here…” be changed to “In the current study,…?

**RESPONSE 20:** It has been removed from this sentence.

**COMMENT 21:** Line 314: Should “and chickensTMUV, and…” (note the lack of a space after chickens) be changed to “and chickens, and…?”

**RESPONSE 21:** It has been corrected as “and chickens, and…” (Page 19, line 337).

**COMMENT 22:** Line 316: Why is “duck” capitalized?

**RESPONSE 22:** It has been corrected as “duck” (Page 19, line 340).

**Reviewer #1:**

**COMMENT 1:** The study was comprised of a loose assemblage of field sampling using methods more or less specific for night-biting zoophagic species. The objectives for using this approach were not clearly described. This sampling was unexpected considering the recent outbreaks of dengue and chikungunya transmitted by anthropophagic day-biting Aedes that are best sampled by other methods. The sporadic nature of the sampling makes the MIR estimates collection specific and not a representation of overall risk.

**RESPONSE 1:** Thanks for your comment.

(1) We have used a human-baited double net for collecting anthropophagic day-biting Aedes and lamp-trapping or animal tent-trapping techniques for night-biting mosquitoes or biting midges. This information has been added to the method section, lines 90-93.

(2) We agree that “The sporadic nature of the sampling makes the MIR estimates collection specific and not a representation of overall risk.” We have modified it as a collection-specific risk as per your suggestion.

**COMMENT 2:** The handling of the collections in the field was not clearly indicated. Were the specimens frozen immediately after capture using dry ice or where the specimens transported alive to a regional laboratory. It was not clearly stated where the laboratory work and virus isolations were done.

**RESPONSE 2:** We appreciate the reviewer's comment.

The specimens were frozen immediately after being captured using dry ice at the collection site and then transported to the vector laboratory of the Hainan Provincial Center for Disease Control and Prevention (CDC) through a cold chain. The mosquito/midge morphological identification, sample grouping, sample homogenization, and RNA detection were conducted in Hainan Provincial CDC. A portion of the homogenization supernatants for the sample were transported to the Wuhan Institute of Virology, Chinese Academy of Sciences, for virus isolation in a biosafety laboratory. This information has been added to the method section, page 5, lines 95-104.

**COMMENT 3:** The diagnostic and virological methods seem adequately presented and represent the 'heart' of the paper. However, there was no indication where the isolates were stored and whether the sequences were deposited in GenBank -- usually deposition references are included.

**RESPONSE 3:** The virus isolates have been stored in the National Virus Resource Center of the Wuhan Institute of Virology, CAS. Information about sequences has been deposited in GenBank or China National Center for Bioinformation, and the accession numbers are listed in Table1.

**COMMENT 4:** The results were clearly presented. It was not evident how many species were represented within the group labeled 'Culex sp.' nor was it explained why these mosquito specimens were not identified to species.

**RESPONSE 4:** We thank the reviewer for this comment.

When we collected the samples, *Culex gelidus* and *Culex tritaeniorhynchus* were mixed because some dorsal scales of *Culex gelidus* were missing, making the morphological identification difficult. So, the “*Culex spp.*” samples contained *Culex tritaeniorhynchus* and *Culex gelidus*.

**COMMENT 5:** The authors' conclusions were well presented and the public/veterinary importance clearly indicated. A case could be made for using a One Health approach to monitoring arbovirus activity on Hainan in a more consistent manner to determine outbreak risk.

**RESPONSE 5:** We appreciate the reviewer for the comment.

**COMMENT 6:** Although well-prepared, I have recommended 'minor' revision to improve the presentation [see attached file for editorial suggestions and minor comments].

**RESPONSE 6:** We appreciate the reviewer for suggestions and edits for our manuscript.

**COMMENT 7:** As mentioned earlier, the main weakness of the study is lack of consistency of sampling in time and space. The collection data are therefore confounded and can't really used to present clearly defined estimates of arbovirus risk. The data do indicate the presence and continued persistence of some zoonotic viruses known to be active in mainland China. Despite this, the data are unique for Hainan, a very different part of China.

**RESPONSE 7:** We agree with it. The limitation of the study is the lack of consistency in sampling in time and space. So, the estimates of arbovirus risk can only be a collection-specific risk but not an overall risk. We have added this statement in the discussion section. Page 10-11, lines 199-202.

**Reviewer #2:**

**COMMENT 1:** The methods used to generate the data presented in this manuscript are clearly presented and all of the information needed to reproduce the results is present.

**RESPONSE 1:** We appreciate the reviewer for the comment.

**COMMENT 2:** The results are mostly clearly presented. Figures and tables are good, although the resolution in the map figure made it hard to read (hard, but not impossible).

**RESPONSE 2:** We have updated the Fig. 1.

**COMMENT 3:** The conclusions are justified.

**RESPONSE 3:** We appreciate the reviewer for the comment.

**COMMENT 4:** Minor edits noted:

Line 63: Sever should be severe.

Line 64: animal disease should be animal diseases.

Line 66: commas after the words island and zones.

Resoluion on figure 1 is poor.

Lines 167 - 171: This sentence is confusing. Consider rewording so that it's clear the total number of PCR positive pools. This is minor, but I had to read the sentence three times to understand it.

Table 1. Column heading should read 'habitat,' not 'habitant.'

**RESPONSE 4:** We have carefully reviewed and corrected the errors according to your suggestions. The Fig. 1 has been updated. For Lines 167-171, we are sorry for this inaccurate description; we have corrected the sentence as “Seventeen pools tested positive by RT-PCR, including fifteen mosquito pools: nine for Japanese encephalitis virus (JEV), three for Tembusu virus (TMUV), and three for Getah virus (GETV). Additionally, two biting midge pools were positive, one for Oya virus (OYAV) and one for Bluetongue virus (BTV) (Table 1)” in page 10 lines 184-187.

**COMMENT 5:** Wu and colleagues present a study of mosquito- and midge-borne arboviruses in Hainan Island China. This is a surveillance-based study: arthropods were captured, identified, pooled and screened for viral RNA. Pools containing viral RNA were inocluated onto cells and viruses isolated. Nucleotide sequences were determined and phylogenetic analyses conducted. In addition, the mutations that differ from previously circulating viruses, and those that may confer interesting phenotypes are described.

**RESPONSE 5:** We appreciate the reviewer for the comment.

**COMMENT 6:** The manuscript reports isolation of several viruses, some are first records for this island. Thus, there is new information presented in this manuscript, although much of what is reported is confirmatory - we already knew that JEV was present on the island, for example.

**RESPONSE 6:** We appreciate the reviewer for the comment.

**COMMENT 7:** Moreover, this is a straightforward and well done manuscript that will be of interest to readers of PNTD. My sole somewhat significant critique of the work is that I don't think that time and space have been well integrated into the analyses that are presented. Upon first reading, it was striking that the study ran from 2019 to 2023, but the only positive virus detections were recorded in 2022 and 2023. I had to dig into the supplement to learn that only a few pools were collected in 2019 and 2021, and none were collected in 2020. The lack of consistent sampling effort should be noted in the main text so that readers understand that the data collected may not be well suited to distinguishing fluctuations in epidemic or zoonotic risk during the period of observation. It would be helpful if the authors could comment on this in the discussion - they do a nice job of covering the genetic data, but the epidemiological data is handled less well.

**RESPONSE 7:** We agree with it. The lack of consistent sampling effort is one of the limitations of our study, so the estimates of arbovirus risk can only be a collection-specific risk but not an overall risk. This has been added to the discussion section (Page 10-11, lines 199-202). In addition, we have modified the Fig. 1 to add information for sample numbers in each year at each city. It will make the reader know the sample collection more clearly.

Fig. 1. Sampling locations for mosquitoes and Culicoides biting midges between 2019 and 2023 on Hainan Island, China. HK: Haikou city, WC: Wenchang city, QH: Qionghai city, WN: Wanning city, LS: Lingshui county, TC: Tunchang county, QZ: Qiongzhong county, BT: Baoting county, SY: Sanya city, DZ: Danzhou city, BS: Baisha county, CJ: Changjiang county, LD: Ledong county, LG:Lingao country, CM:Chengmai country, DA:Dingan country, DF:Dongfang city. (Note: Blank cities refer to areas that have not been sampled. In the stacked bars, the left Y-axis represents the Log10 values of collected mosquitoes, with different colors indicating various mosquito species.

The source of the basemap shapefile was from Resource and Environmental Science Data Platform (https://www.resdc.cn/DOI/doi.aspx?DOIid=121)[19]. The visualization for the map was constructed through R v4.3.3 (https://www.r-project.org/ ) package ggplot2 3.5.1(https://ggplot2.tidyverse.org/）. The images in Fig1 created in BioRender. Huang, D. (2024) BioRender.com/u66x097).

**Reviewer #3:**

**COMMENT 1:** The current manuscript lacks a clearly defined hypothesis and articulated aims. The authors should improve the abstract and introduction sections by including a specific scientific aim. Using epidemiological or historical data to frame this aim would enhance the study's context and relevance.

**RESPONSE 1:** We thank the reviewer for this comment.

In the early 1990s, a large-scale survey for arbovirus in vectors was conducted in Hainan. However, in the past 30 years, with climate change, urbanization, and the increasing population and trades, whether the arbovirus spectrum changed on this Island is unclear. We screened arboviruses in mosquitoes and biting midges from 2019 to 2023 to assess the presence of arbovirus and the genetic evolution currently circulating on this Island. This information has been added to the abstract section on page2-3, lines 40-45.

**COMMENT 2:** Furthermore, the manuscript would benefit from additional background information on the sampling collection process. Detailed information about the environments selected at each site, the criteria for site selection, the number of traps set per site, and the duration of the sampling effort in days should be included. It is important to specify whether the sampling effort was consistent across all collection sites.

**RESPONSE 2:** We thank the reviewer for the comments.

(1) Detailed information such as geography, species, collection date, and habitat types on sample collection were listed in Table S2.

(2) The lack of consistent sampling effort is one of the limitations of our study, so the estimates of arbovirus risk can only be a collection-specific risk but not an overall risk. This has been added to the discussion section (Page 10-11, lines 199-202). We have modified the Fig. 1 to add information for sample numbers for each year at each city. It will make the reader know the sample collection more clearly.

**COMMENT 3:** Additionally, the authors should provide detailed information on the handling and transportation of the mosquitoes. Clarify whether the mosquitoes were identified in the field or the lab and describe how the cold chain was maintained during the identification process and transportation.

**RESPONSE 3:** We thank the reviewer for this comment. We have used a human-baited double net for collecting anthropophagic day-biting Aedes and lamp-trapping or animal tent-trapping techniques for night-biting mosquitoes or biting midges. The specimens were frozen immediately after being captured using dry ice at the collection site and then transported to the vector laboratory of the Hainan Provincial Center for Disease Control and Prevention (CDC) through a cold chain. The mosquito/midge morphological identification, sample grouping, sample homogenization, and RNA detection were conducted in Hainan Provincial CDC. A portion of the homogenization supernatants for the sample were transported to the Wuhan Institute of Virology, Chinese Academy of Sciences, for virus isolation in a biosafety laboratory. This information has been added to the method section, page 5, lines 95-104.

**COMMENT 4:** Please clarify whether you inoculate mosquito homogenates only from those with PCR-positive results or from all homogenates generated in the study.

**RESPONSE 4:** All homogenates generated in the study have been inoculated into C6/36 cells, BHK-21 cells, and Vero-E6 cells for virus isolation. This information has been added to the method section, page 6, lines 127-128.

**COMMENT 5:** The authors could provide a more detailed description of the most important mosquito species and midges, focusing not only on general characteristics but also specifically in relation to the sampling collection. They do not need to describe all species per site, just the most frequent or medically important ones. Additionally, include the 95% binomial confidence interval in the results for mosquitoes positive for each virus, as it is important to convey the uncertainty around the central estimates.

**RESPONSE 5:** We thank the reviewer for the valuable suggestion.

(1) We have removed the mosquito species with low numbers and retained the most frequent or medically important ones as “The mosquitoes were comprising *Culex tritaeniorhynchus* (34.96%), *Aedes albopictus* (16.51%), *Armigeres subalbatus* (9.78%), *Culex gelidus* (5.31%) and unidentified *Culex spp.* (contained *Cx. tritaeniorhynchus* and *Cx. gelidus, Cx. gelidus* and *Cx. tritaeniorhynchus* were mixed because of some dorsal scales *Cx. gelidus* were missing, making them morphologically indistinguishable from *Cx. tritaeniorhynchus*) (26.69%) (Fig. 1 and S2 Table).” In page 8 lines 160-167.

(2) Additionally, the 95% binomial confidence interval in the results for mosquitoes positive for each virus has been added in Table 1.

**COMMENT 6:** The authors are required to rewrite the discussion section and make adjustments once a clear aim is stated. They need to better discuss how their observations align with the current epidemiology in Hainan Island. If these viruses are not currently being detected in the region, the authors should explore possible reasons

for this discrepancy. Based on their results, the authors should provide suggestions for improving laboratory and epidemiological surveillance in the region.

**RESPONSE 6:** We thank the reviewer for the comment. The discussion section has been rewritten according to your suggestion. Page 17-22 lines 283-389.

**COMMENT 7:** The figures clearly match the results, and no changes are required at this stage.

**RESPONSE 7:** We thank the reviewer for the comment.

**COMMENT 8:** The study presents a comprehensive survey of arboviruses in mosquitoes and biting midges on Hainan Island, China, conducted between 2019 and 2023. Utilizing RT-PCR assays, the researchers aimed to detect the presence, distribution, and infection rates of mosquito-borne pathogens. The study also employed cell inoculation and high-throughput sequencing to isolate viruses and assemble full viral genomes, followed by phylogenetic analysis to determine viral genotypes and evolutionary relationships.

**RESPONSE 8:** We thank the reviewer for the comment.

**COMMENT 9:** The survey collected a substantial number of specimens, including 32,632 mosquitoes and 21,000 biting midges from 14 out of 18 cities/counties on the island. Notable findings include the detection of Japanese encephalitis virus (JEV), Tembusu virus (TMUV), Getah virus (GETV), Oya virus, and Bluetongue virus (BTV). The study successfully isolated and sequenced several virus strains, contributing valuable data to the understanding of arbovirus circulation on Hainan Island.

**RESPONSE 9:** We thank the reviewer for the comment.

**COMMENT 10:** Specific comments and suggestions:

Introduce a clear scientific aim and hypothesis.

Include detailed descriptions of the most frequent or medically important mosquito species and midges at the sampling collection in the result section.

Provide comprehensive background information on the sampling methodology and environment.

Detail the handling and transportation procedures for the specimens in the methodology section.

Incorporate the 95% binomial confidence intervals in the results section.

Revise the discussion to align observations with current epidemiological data and suggest improvements for surveillance based on findings.

**RESPONSE 10:** We thank the reviewer for the critical comments and helpful suggestions.

(1) In response to comment 1 we have added the hypothesis and articulated aims as “These results stress the need to develop adequate surveillance plan measures to better control the public health threat of arboviruses carried by mosquitoes and biting midges in local regions.” and “In the early 1990s, the large-scale survey for arbovirus in vectors had been only conducted in Hainan. In the past 40 years, with climate change, urbanization, and the increasing population and trades, whether the arbovirus spectrum changed on this Island is unclear. We screened arboviruses in mosquitoes and biting midges during 2019–2023 to assess the arbovirus presence and the genetic evolution currently circulating in this Island.” in lines 36-38 and lines 40-45.

(2) We have removed the mosquito species with low numbers and retained the most frequent or medically important ones.

(3) Detailed information about geography, species, collection date, and environments on sample collection were listed in Table S2.

(4) The 95% binomial confidence interval in the results for mosquitoes positive for each virus has been added in Table 1.

(5) We added the current epidemiological data and suggested improvements for surveillance based on the findings. As “Human cases of JE have rarely been reported on Hainan Island in recent years due to JE vaccination programs. From 1991 to 2018, Hainan reported few or no local dengue cases for over 20 years. However, since September 2019, the number of reported local dengue cases has increased annually. The animal diseases associated with GETV, TMUV, and BTV have been endemic in mainland China, but there are currently no reports of this animal disease outbreak in Hainan. Our detection of JEV, GETV, TMUV, and BTV in vectors suggests an alarming increase in arbovirus diversity and a heightened risk of infection on Hainan Island.” in lines 366-374.
