# Supplementary material for: Detection and evolutionary characterization of arboviruses in mosquitoes and biting midges on Hainan Island, China, 2019–2023
Source: PLoS Negl Trop Dis. 2024 Oct 31;18(10):e0012642. doi: 10.1371/journal.pntd.0012642 (PMC11556698; doi:10.1371/journal.pntd.0012642)
Supplement: Attachment — Submitted filename: Response to Editor-1011.docx [file pntd.0012642.s008.docx]

**Cover Letter in Response to Editor’s Comments**

Dear Editor,

Thank you for recognizing our work. We have thoroughly revised the manuscript based on your comments. Response to each comment is listed below. For your convenience, we have marked the response text in blue.

Thanks for your consideration.

Sincerely,

Han Xia, Ph.D

Professor

Key Laboratory of Virology and Biosafety

Wuhan Institute of Virology, Chinese Academy of Sciences

Wuhan, 430207, China

Tel: 86-027-87998215

E-mail: hanxia@wh.iov.cn

**COMMENT 1:** Lines 27 and 34 (and many others): Should numbers less than 10 be written out (as on line 34) or should numerals be used (as on line 27)? For PLoS NTD, I believe that either is acceptable, but you need to be consistent. Note, units of measurement are always numerals, i.e., 3 days. Because you have written out nearly all of the numbers <10 (which is the method that I prefer), I suggest that you go through the manuscript and write out all non-measurement numbers <10.

**RESPONSE 1:** We thank the editor for the critical comments and helpful suggestions. We have reviewed the manuscript and written out all non-measurement numbers <10.

**COMMENT 2:** Line 98: I am not sure what you mean by “through cold chain.” Were the specimens maintained on dry ice, liquid nitrogen, kept frozen using regular ice, maintained in a refrigerated container, etc.? There are differences for virus isolation. Please be a little more specific.

**RESPONSE 2:** Thanks for pointing out this. The specimens were transported through a cold chain with dry ice and then maintained in a refrigerated container. This information has been added to the method section. (Page 5, lines 105-106)

**COMMENT 3:** Lines 160-164: The sum of these percentages is 93.3. Obviously, some other species (e.g., Ae. cinereus, Ae. vexans, Anopheles spp., etc.) were collected in limited numbers. You should add, “and other species in limited numbers (6.7%)” just before “(Fig.1 and S2 Table).”

**RESPONSE 3:** It has been added as “and other species in limited numbers (6.7%)” before “(Fig.1 and S2 Table)” on page 8, lines 165-166.

**COMMENT 4:** Lines 214-224: What about the other six strains where the actual virus was not isolated? You mentioned that the entire E segment was present. Did you sequence it?

**RESPONSE 4**: Yes, the entire E segment was sequenced. Since it is not a whole genome, we did not submit the sequence to NCBI.

**COMMENT 5:** Lines 218-220: Are you only referring to the “latter isolate,” i.e., the one from Qionghai, or are you referring to all three isolates being within GI? If it is only the latter one, it should be, “The latter isolate clustered…” and if it is all three, it should be, “These isolates clustered…” What about the other 6 JEV detected; were they also GI?

**RESPONSE 5**: It has been corrected as “These isolates clustered together within GI (Fig. 2A)”. (Page 11, lines 219-220).

The other 6 JEVs detected were also clustered GI based on the envelope protein （E）gene of JEV (S Figure).

S Figure. Phylogenetic tree constructed based on the envelope protein （E）gene of Japanese encephalitis virus (JEV). The red star indicates the detected JEV or isolate in this study.

**COMMENT 6:** Line 290: By “this species” do you mean Culex tritaeniorhynchus and Culex gelidus, or just Culex tritaeniorhynchus. I assume just Culex tritaeniorhynchus, so you might want to change the “this species” to “Culex tritaeniorhynchus.”

**RESPONSE 6**: It has been changed from “this species” to “*Cx. tritaeniorhynchus*” on page18, line 291.

**COMMENT 7:** Line 296: Here it says that GIII was replaced by GI in the last 20 years. However, if GIII was still present in 2008, that was only 16 years ago and only 11 years before the start of the current study. Should 20 be changed to 15?

**RESPONSE 7**: It has been changed from “in the last 20 years” to “in the last 15 years” on page 18, line 297.

**COMMENT 8:** Lines 374: Yes, your detection of these viruses in potential vectors indicates that the diseases caused by these viruses are likely present on Hainan Island. As I have often said, if you do not look, the disease is not present. You might want to add, “However, the lack of reported disease may be due to a lack of recognition, rather than due to the absence of disease.” after Hainan Island and before DENV…

**RESPONSE 8**: It has been added “However, the lack of reported disease may be due to a lack of recognition, rather than due to the absence of disease.” in page 22, lines 374-375.

**COMMENT 9:** Line 27: as GETV was detected in only 1 city, it should be “from one city/county with…” As you stated that Oya and BTV were detected in Wanning City, why not indicated that GETV was detected in Qionghai, i.e., “from Qionghai with…”

**RESPONSE 9**: It has been corrected as “from Qionghai city with…” on Pag 2, line 27.

**COMMENT 10:** Lines 45-47: I might modify this sentence to, “Except for arboviruses such as JEV, GTEV, and OYAV, which have been previously reported circulating on Hainan Island, TMUV and BTV were detected for the first time during this survey.”

**RESPONSE 10:** It has been changed from “Except the arbovirus such as JEV, GTEV, and OYAV, which has been reported circulating on Hainan Island, TMUV and BTV were detected for the first time during the survey.” to “Except for arboviruses such as JEV, GTEV, and OYAV, which have been previously reported circulating on Hainan Island, TMUV and BTV were detected for the first time during this survey.” on (Page 3, lines 49-47.)

**COMMENT 11:** Lines 79-80: DENV was already established on line 62 and JEV on line 63.

**RESPONSE 11:** We have carefully reviewed and corrected this error based on your suggestion.

**COMMENT 12:** Line 96: “in the” collection site should be “at the” collection site.

**RESPONSE 12:** It has been corrected as “at the”. (Page 5, line 96).

**COMMENT 13:** Line 104: “…in biosafety laboratory” should be “in a biosafety laboratory.”

**RESPONSE 13:** It has been corrected as “in a biosafety laboratory”. (Page 5, line 106).

**COMMENT 14:** Line 127: This should be, “…study were inoculated…” and there should be a “,” after the “cells” of the BHK-21 cells.

**RESPONSE 14:** It has been corrected as “…study were inoculated…” and the “,” have been added after the “cells” of the BHK-21 cells on page 6, line 130.

**COMMENT 15:** Line 160: Change “were comprising…” to “consisted of…”

**RESPONSE 15:** It has been changed from “were comprising…” to “consisted of…” on page 8, line 162.

**COMMENT 16:** Line 181: Check the spacing around the “.” Just before “The…”

**RESPONSE 16:** It has been checked and deleted a space around the “.” before “The…”. Page 10, line 183.

**COMMENT 17:** Line 184: The “fifteen mosquito…” should be “15 mosquito…”

**RESPONSE 17:** It has been changed from “fifteen mosquito…” to “15 mosquito…” on page10, line 186.

**COMMENT 18:** Line 195: There needs to be a space between “(0-4.0)” and “per…”

**RESPONSE 18:** A space has been added between “(0-4.0)” and “per…”. Page 10, line 197.

**COMMENT 19:** Line 200: “Aedes” should be on italics.

**RESPONSE 19:** It has been corrected as “*Aedes*”.

**COMMENT 20:** Lines 204-205: The samples were not inoculated with the cells, the cells were inoculated with the samples. Should this be, “…were inoculated onto C6/36…?” Also, because you are writing out most number <10, this should be “nine viral…” and on the next line, it should be “one strain…”

**RESPONSE 20:** It has been corrected as “In total, 765 pools of mosquito and biting midge homogenates were inoculated onto C6/36, BHK-21, and Vero-E6 cells, and a total of nine viral isolates were obtained, of which eight strains were able to cause CPEs in C6/36 cells, and one strain (HN-WN22-Cu-18) caused CPEs in BHK-21 cells.” (Page11, lines 206-209).

**COMMENT 21:** Line 284: Change “warnings, not only the RT-PCR…” to “warnings, RT-PCR…,” i.e., remove the “not only the” as you used all three methods.

**RESPONSE 21:** It has been corrected as “To strengthen epidemiological investigations of arboviruses and meet the need for early disease warnings, RT-PCR detection, virus isolation, and deep sequencing were used in combination in our study.” (Page18, lines 285-287).

**COMMENT 22:** Line 289: “Contained” should be “contained” without the capital “C.” Also, as Cx. has been established, these should be Cx. tritaeniorhynchus and Cx gelidus” (note, they should be in italics, but this program does not allow me to use italics.

**RESPONSE 22:** It has been corrected as “The detection of JEV in *Cx. tritaeniorhynchus* and *Culex* spp. (contained *Cx. tritaeniorhynchus* and *Cx. gelidus*) pools from five cities and counties in Hainan Island confirmed that *Cx. tritaeniorhynchus* was the main vector of JEV, and it was widespread in Hainan.” (Page 18, lines 290-293)

**COMMENT 23:** Line 323: There should be a “,” after “flavivirus” and before the and.

**RESPONSE 23:** It has been added as “,” after “flavivirus” and before the and. Page 20, line 325.

**COMMENT 24:** Line 351: There should be a space between “1964” and “[47]…”

**RESPONSE 24:** A space has been added between “1964” and “[47]…”. Page 21, line 353.

**COMMENT 25:** Line 356: Shouldn’t “Hainan Provincial Center for Disease Control and Prevention…” be Hainan Provincial CDC…?”

**RESPONSE 25:** It has been corrected as “Hainan Provincial CDC…”. Page 22, line 367.

**COMMENT 26:** Line 383-384: As “years” is a unit of measurement, it should be “3 years…” and “1 or 2 years.”

**RESPONSE 26:** It has been corrected as “3 years…” on page 23, lines 385.

**COMMENT 27:** Line 384: It is not despite the inconsistencies that the data may not be well suited, it is because of the inconsistencies. I would modify the sentence to, “Because of the inconsistencies in…”

**RESPONSE 27:** It has been changed from “Despite inconsistencies” to “Because of the inconsistencies in” on page 23, line 386.

**COMMENT 28:** Line 463: Because “spp.” is not an actual species name, it should not be in italics.

**RESPONSE 28:** It has been corrected as “spp.” on page 26, line 466.

**COMMENT 29:** Line 512: Culex tritaeniorhynchus should be in italics.

**RESPONSE 29:** It has been corrected as “*Culex tritaeniorhynchus*”. Page 27, line 515.

**COMMENT 30:** Line 528: Japanese Encephalitis should be Japanese encephalitis without the capital.

**RESPONSE 30:** It has been corrected as “Japanese encephalitis” on page 28, line 531.

**COMMENT 31:** Line 587: Because “blue” is not a proper noun, it should not be capitalized.

**RESPONSE 31:** It has been corrected as “blue foxes” on page 29, line 590.
